# Correlation between proprioception, functionality, patient-reported knee condition and joint acoustic emissions

**DOI:** 10.1371/journal.pone.0310123

**Published:** 2024-11-06

**Authors:** Liudmila Khokhlova, Dimitrios Sokratis Komaris, Brendan O’Flynn, Salvatore Tedesco

**Affiliations:** 1 Insight Centre for Data Analytics, Tyndall National Institute, University College Cork, Cork, Ireland; 2 Aston University, Birmingham, United Kingdom; King Khalid University, SAUDI ARABIA

## Abstract

Non-invasive assessment of joint status using acoustic emissions (AE) is a growing research area that has the potential to translate into clinical practice. The purpose of this study is to investigate the correlation of the knee’s AE with measures of proprioception, self-assessment, and performance, as it can be hypothesised that, AE parameters will correlate with joint function metrics due to AE being recorded during interaction of the articular surfaces. Threshold to detect passive motion (TTDPM), Knee Osteoarthritis Outcome Scores (KOOS) and 5 times sit-to-stand test (5STS) were collected from 51 participant. Knee AE were recorded during cycling with 30 and 60 rpm cadences using two sensors in different frequency ranges and three modes of AE event detection. Weak (0.297, p = 0.048) to moderate (0.475, p = 0.001) Spearman’s correlations were observed between longer 5STS time and AE parameters (i.e. higher median absolute energy, signal strength, longer AE event rise time and duration). Similarly, AE parameters shown correlation with lower KOOS, especially in the “Function in Sports and Recreation” and “Activities of Daily Living” subscales with correlation coefficients for higher median amplitude up to 0.441, p = 0.001 and 0.403, p = 0.004, respectively. The correlation with the TTDPM was not detected for most of the AE parameters. Additionally, a lower frequency sensor and 60 rpm cadence AE recordings showed higher correlations. Considering that this study included subjects from the general population and the number of participants with KOOS <70 was relatively small, higher correlations might be expected for clinically confirmed OA cases. Additionally, different ICCs might be expected for alternative signal parameters and proprioception assessment methods. Overall, the study confirms that AE monitoring offers an additional modality of joint assessment that reflects interaction between cartilage surfaces and can complement orthopaedic diagnostics, especially in the context of remote monitoring, drug testing, and rehabilitation.

## Introduction

Joint disorders, in particular osteoarthritis, are widespread in the older population, with the global prevalence of knee osteoarthritis estimated at 16%, and an incidence rate of 203 per 10,000 person-years [1]. Taking into account the high obesity rates and the ageing population, known risk factors of osteoarthritis [2], the numbers are predicted to increase in the future decades [3, 4]. Currently, the knee condition is often assessed by radiographic or MRI findings, based on scales such as the Kellgren-Lawrence radiographic classification scheme [5], or the Whole-Organ Magnetic Resonance Imaging Score (WORMS) [6]. Imaging technologies have proven to be a valuable tool in orthopaedics but are often inaccessible due to equipment size and complexity, need for trained personnel, and high costs, making them impractical for remote or continuous monitoring like at-home rehabilitation. At the same time, weak correlations have been found between clinical indicators such as patient-reported function and radiographic or MRI findings (specifically, tibiofemoral cartilage loss [7, 8] and the pattern changes in bone marrow oedema [8]). In parallel, patient-reported outcome measures (i.e., patient self-assessments regarding health, symptoms, quality of life, functionality, etc. [9]) are extremely important in clinical practice, as ultimately, a successful treatment is determined by the patients’ satisfaction. With the rise of personalised medicine, patient outlook gains attention in medical device and drug development, with regulatory bodies like the U.S. Food & Drug Administration and the European Medicines Agency both recognising and encouraging the use of patient reported measures [10]. Considering the above, there is an interest in approaches to joint condition assessment that can provide objective metrics, which correspond to recommended self-reported measures and are suitable for applications outside of the current scope of traditional imaging techniques, such portable or wearable applications.

One such alternative is acoustic emission (AE) monitoring [11], a non-destructive testing technology commonly used to detect the defects in a variety of structures [12]. AE monitoring is based on the recording of transient elastic waves generated within a material or structure and caused by deformation, friction, corrosion etc. In the orthopaedic context, the method has been successfully used to distinguish healthy from osteoarthritic knees [13] identify implant loosening and injury [14], or locate cartilage lesions [15]. AE parameters have been associated with MRI [16] and radiographic [13] findings, indicating the clinical potential of AE monitoring. Contrary to most imaging technologies and chemical biomarkers of osteoarthritis, AE monitoring provides information that is directly related to knee function, as AE are recorded during movement and often under a load [14], and can potentially be recorded during extended periods. With small to moderate association between patients’ functional performance with MRI findings [17], AE monitoring can potentially provide an additional objective measure associated with performance metrics or self-reported outcomes, especially considering a different approach to signal recording. Moreover, AE monitoring does not require complex equipment and can offer a low-cost complementary diagnostic modality suitable for home and wearable monitoring solutions [18].

Proprioception is another aspect that correlates to joint functionality [19], degradation of the articular cartilage due to osteoarthritis [20], and meniscal abnormalities [21]. Joint injuries and disorders, and in particular osteoarthritis, can cause articular cartilage damage and changes in the structure around the joint (e.g., capsule, muscles, and tendons) where the mechanoreceptors are located. Thus, poor proprioception is associated with cartilage damage, which in turn was shown to result in abnormal AE output [15, 22]. Moreover, increased age is also associated with a decline in proprioceptive function [23] and changes in the knee’s AE [24], further indicating a possible correlation of AE outputs with proprioception.

Thus, the objective of this study is to investigate the relationship between knee AE parameters and measures of functionality and satisfaction, specifically correlation with performance, patient reported symptoms, daily function, quality of life, and proprioception. We hypothesize that an increased number of recorded AE events, their amplitude, duration, energy and other parameters, will be associated with worsened joint function due to the active engagement of the joint and, specifically, the cartilaginous articular surfaces during motion. A secondary outcome of this study is the comparison of two AE sensors with different frequency ranges and multiple AE event parameters to determine the optimal sensor configuration.

## Materials and methods

The study was conducted according to the guidelines of the Declaration of Helsinki and was approved by the Clinical Research Ethics Committee of the Cork Teaching Hospitals at the University College Cork (Ref. number: ECM 4 (e) 17/05/2022 & ECM 3 (kkk) 17/05/2022), and written informed consent was obtained from all participants involved in the study. The recruitment period of the study—05.06.2022–30.12.2022.

A total sample size of 47 participants was determined by a two-sided sample size calculation, with α = 0.05 (level of significance) and β = 0.20 (1- β, power of the study), and an expected correlation coefficient of 0.4, based on the previously observed effect sizes and correlations of MRI measures with the self-reported outcome scores (KOOS) and functional tests (r = 0.15–0.36 [7] and η^2^ = 0.087–0.222 [17], respectively). Fifty-one participants (Table 1) were recruited via word of mouth and email advertisement. The sample includes older (50–75 years old, 12 females, 14 males) and younger adults (18–35 years old, 12 females, 13 males) that were consecutively recruited to ensure various KOOS scores [25]. The exclusion criteria included factors that can affect quality of AE recording or participant’s safety: BMI above 40, acute injuries, inflammation, oedema, significant hip and ankle comorbidities and any other general conditions that might prevent safe exercise execution.

**Table 1:** Sample anthropometrics and demographics.

Median Age (IQR^a^), years	Mean Height (SD^b^) cm	Mean Weight, kg	Mean BMI, kg/m2	Median KOOS (IRQ)	Median 5STS (IQR), s	Median TTDPM (IQR), deg
50.5 (34)	171.99 (8.53)	75.02 (12.84)	25.33 (3.85)	87.5 (22)	8.02 (3.21)	0.93 (0.64)

^a^ Interquartile range

^b^ Standard deviation

### AE recording

Knee AE were recorded using the USB AE Node system (Mistras, Physical Acoustics) with a sampling rate of 20 Msps and a 20 dB gain. The AE sensors, AE event (hit) registration threshold, and filtering parameters used for the recording are presented in Table 2. The frequency range of recordings is defined by the internal hardware filters of the system [26] and the operating frequency ranges of the sensors [27, 28]. In the existing literature [14] frequency ranges below 35kHZ were more commonly used, however, several studies successfully used frequency ranges of up to 500kHZ. Thus, two sensors covering this frequency range were included in this study to allow for an objective comparison of the results—PK15I and PK3I (Mistras, Physical Acoustics). The sensors are 27 mm in height and 20.6 mm in diameter, weight 51 g. Sensors’ selection was driven by the availability of commercially accessible, low-noise compact solutions.

**Table 2:** AE sensors recording parameters and hit definition threshold.

Sensor	Threshold (fixed), dB	Hardware filter upper, kHz	Hardware filter lower, kHz	Operating frequency range, kHz
PK3I [27]	28	1	200	15–40
PK15I [28]	24	20	500	100–450

The registration of AE events is generally determined by an amplitude threshold and other pre-set parameters, such as hit definition time (the time between threshold crossings that is used to identify the end of an event), peak definition time (determines the peak of the waveform) and hit lockout time (the period during which the system inhibits measurements to avoid registering reflections of an event) [29]. Three modes of hit definition parameters (Table 3) were considered. Firstly, timing parameters were calculated using the techniques provided by the system manufacturer [29], with the optimal peak definition time value was calculated dividing the sensor spacing distance by the speed of the AE wave in the material [30]. Considering the high variability between knee dimensions in the population and the complex joint anatomy that includes tissues with different acoustic properties, the peak detection time was estimated as follows: the speed of sound in the knee was equated to that in the cancellous bone (2140 m/s at 270 kHz [31]) as it comprises the majority of the knee’s joint tissue; as only one sensor was used at a time, the knee diameter was set as the sensor spacing distance, with an average value of 77.2 mm as measured in cadaveric knees [32]. Therefore, the value of peak detection time was calculated to be 36.45 μs, and was rounded up to minimal available preset value—40 μs. The manufacturer also indicates that hit detection time can be set at 20/AC, where AC is the attenuation coefficient (dB/mm) but should be at least twice as long as the peak detection time [29]. With an attenuation coefficient for equal to 295.32 dB/m at 270 kHz [31], the calculated hit detection time is 67.7 μs, but was set to 80 μs to be at least twice the peak detection time. The hit lockout time was set as short as possible, at 40 μs, to allow recording of AE event reflections. Alternatively, recordings were made using values recommended for non-metal composite structures [30], considering bone as composite material: peak detection time of 50 μs, hit detection time of 200 μs, and a longer hit lockout time of 300 μs that allows discarding reflections of the signal. For the third mode, the parameters were set to values previously successfully used to assess joint deterioration and osteoarthritis damage by Shark et al. [16, 24], with peak detection time set to 200μs, hit detection time to 800μs, and hit lockout time to 1000μs.

**Table 3:** AE sensors recording and hit definition parameters.

Mode	Peak definition time, μs	Hit definition time, μs	Hit lockout time, μs
1	40	80	40
2	50	200	300
3	200	800	1000

**Fig 1:**
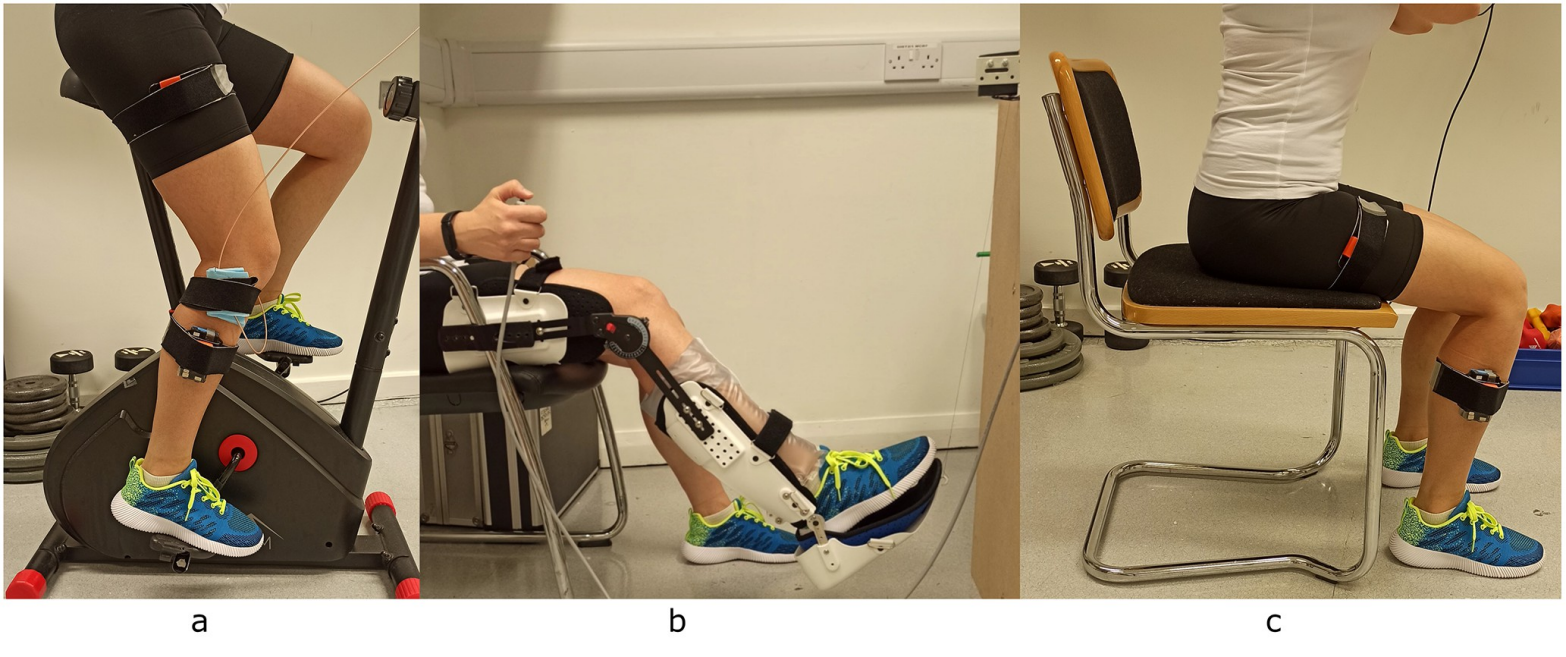
Experimental setup a) knee AE recording b) TTDPM measurement c) functional assessment.

The acquisition method and recording setup closely follows previous works where motion artifact resistant mounting was suggested [33] and test-retest reliability of the method was assessed [34]. The sensor was positioned on the right medial condyle of the tibia as this placement showed the minimum muscular and dynamic artifacts in AE event acquisitions [22, 35]. The sensor was encased in an ethylene-vinyl acetate foam holder and attached to the skin using double-sided skin-safe tape (Arcos, transparent) [34]. Cycling on a stationary bike (Fig 1a) with a fixed cadence was used to excite joint AE, as it showed higher reliability than unconstrained exercises where the influence of movement execution increases the variability in the recordings [34]. Moreover, cycling equalizes the load on the joints of participants with different body weight, as it was associated with AE output in exercises such as the sit-to-stand [36]. Furthermore, cycling is less physically demanding than sit-to-stands and is appropriate for the elders with various disorders [37].

Participants cycled for a minute in each mode and cadence (30 and 60 rpm), and the lowest possible cycling resistance was used to ensure that older and potentially frail participants can execute the exercise easily. The exercise was repeated with two sensors, resulting in twelve records (2 cadences x 3 modes x 2 sensors) for each participant. The notation *Sensor_Mode_Cadence* was used for each type of trial, referring to the low (LF) or high frequency (HF) *sensors*, the *Modes* of Table 3, and 60 or 30 rpm *Cadences*. LF_3_60, for example, denoted trials with the PK31 (LF) sensor, mode 3, at 60 rpm. A metronome and a visual clue of the current cycling speed were used to help participants maintain a stable cadence. Xsens (Xsens Technologies B.V.) inertial measurement units were fixed on the crank of the bike, and the shank and thigh of each participant. An additional custom inertial measurement unit [38] was used to obtain timestamps and synchronise recordings.

### Self-reported outcome measures

Self-reported knee condition was evaluated using the Knee Injury and Osteoarthritis Outcome Score (KOOS), a validated [39] questionnaire that targets symptoms and function of patients with knee injury and osteoarthritis [40], and is one of the most common metrics in clinical practice [41]. The overall KOOS and its five subscales, i.e., “Pain” (KOOS_P), “Other Symptoms” (KOOS_S), “Activities in Daily Living” (KOOS_ADL), “Function in Sport and Recreation” (KOOS_SR), and “Knee-related Quality of Life” (KOOS_QoL) were considered.

### Proprioception testing

Given its complex nature, several types of tests are employed to measure components of proprioception: the threshold to detect passive motion (TTDPM) measures the sense of passive joint movement; other techniques quantify active and passive joint positioning sense, and active movement extent discrimination. While both joint position and TTDPM tests can discriminate between osteoarthritic and healthy controls [20], TTDPM mostly stimulates articular mechanoreceptors and has high conceptual purity [42], whereas the joint position sense and active movement extent discrimination tests stimulate both receptors in joint capsule and muscles. Moreover, such tests are dependent on the participants’ memory, potentially introducing bias [43] and are more suitable for studies focused on conscious perception and voluntary movements. In this study, TTDPM test was considered, as changes in AE are generally associated with articular rather than muscle or neural tissue damage within the knee [14]. TTDPM was previously validated in healthy controls [44, 45] and found to be increasing with age [44].

The proprioception assessment setup was set accordingly to previously reported protocols [23, 46]. Participants were asked to sit on a chair with an attached leg platform (brace, Fig 1b). The rotation axis of the knee was aligned with the rotation axis of the brace before starting each trial. An inflatable brace was used to minimise the sensory input from the skin’s pressure receptors [21].

Over-the-ear headphones with a white noise recording and blindfolds were used to exclude audio-visual cues from the movement of the motor and brace. Alternatively, if a participant was uncomfortable with the blindfold, they were asked to close their eyes and turn their head to the side. The initial angle was set at 45 [23] degrees of knee flexion. A motor was programmed to start lowering a participant’s lower limb by 0.3 degrees per second after a random pause. The detection of motion by the participant was indicated by pressing a handheld button. First three tests introduced the participant to the measurement process, and five more measurements were conducted to measure the average reaction delay. The difference between the starting position and knee angle at the moment when the button was pressed by the participant was used as an indicator of proprioceptive ability.

### Functional assessment

Functional assessment was conducted using the 5 times sit-to-stand test (5STS), as a reliable [47] and easy-to-conduct and less time-consuming test than walking tests evaluating mobility. A straight back chair with a height of 49 cm was used, and the body worn IMUs provided accurate measurements of execution time (Fig 1c).

### Data processing

AE were logged using the AEwin software (Mistras, Physical Acoustic). All sensor recordings were synchronised and further analysed in MATLAB (Mathworks). Specifically, IMU data were used to segment the cycling records into rotations and measure the duration of the 5STS test. When processing cycling records, the first rotation and all rotations that differed from the assigned cadence by more than 20% were excluded; for each record, 40 and 20 rotations that met the cadence criteria of 60 and 30 rpm, respectively, were considered.

The records were filtered to include only AE events with a duration greater than 1μs, thus discarding hits that potentially originated from motion and friction artifacts. Such filtering techniques based on duration and/or amplitude are commonly used in non-destructive AE testing [48, 49]. The following AE hits parameters were further analysed: mean number of hits per rotation; median AE hit amplitude, dB; median hit duration, μs; median time to peak of the hit (rise time), μs; median signal strength, pV-s; and median absolute energy, attoJoules. These parameters showed the highest reliability scores in a preliminary test-retest evaluation study for the same setup [34] or were most indicative of cartilage damage in a pilot cadaver study [22]. Moreover, using the median values allows for a robust measure of skewed data and in the presence of outliers. Additionally, the number of hits above threshold previously used in literature 32 [50] and 36 dB [16] were computed.

Considering that exercise execution can affect recorded AEs, the movement smoothness for 30 rpm (i.e., LF_1_30) and 60 (i.e., LF_1_60) rpm recording was assessed using the log dimensionless jerk for 10 consecutive repetitions within the assigned cadence ±20%. The shank IMU’s free acceleration signals were filtered with a low-pass, second-order, zero-phase shift Butterworth filter with a cut-off frequency of 6 Hz and log dimensionless jerk was calculated according to [51].

### Statistical analysis

The statistical analysis was performed using IBM SPSS Statistics v.28. Standard descriptive statistics such as means, medians, standard deviations and interquartile ranges were calculated for all measures (KOOS, 5STS, and TTDPM) and AE parameters and presented in the S1 Appendix. Normality of the data was assessed using the Shapiro-Wilk test. Spearman’s coefficient was used to assess the correlations between 5STS time, KOOS, proprioception, log dimensionless jerk and medians of AE parameters, to minimize the influence of outliers and heavy-tailed data distributions [52] (e.g., AE event amplitudes above a specific threshold). All data points were included in the analysis. Scatter plots were visually assessed to confirm monotonic relationship between variables. Significance was set at *P* = 0.05. Correlation strength was classified as weak (0.1–0.3), moderate (0.4–0.6), or strong (0.7–0.9) [53].

## Results

Twenty AE recordings out of 612 and 6 comparison measures (3 for proprioception test and 3 for 5STS) were excluded from analysis due to being incomplete or erroneous (S1 Appendix). For participant 8 (record LF_3_60), 33 instead of 40 repetitions were included, as the remainder of the repetitions did not fall within 20% of the requested cadence. Similarly, for participant 22, 38 repetitions were considered in the LF_1_60, LF_2_60, and HF_1_60 recordings. Full tables, including weak or non-significant correlations, p-values of Spearman’s correlations and selected scatter plots (ρ>0.4) are included in the S3 Appendix.

### Self-reported assessment

**Fig 2:**
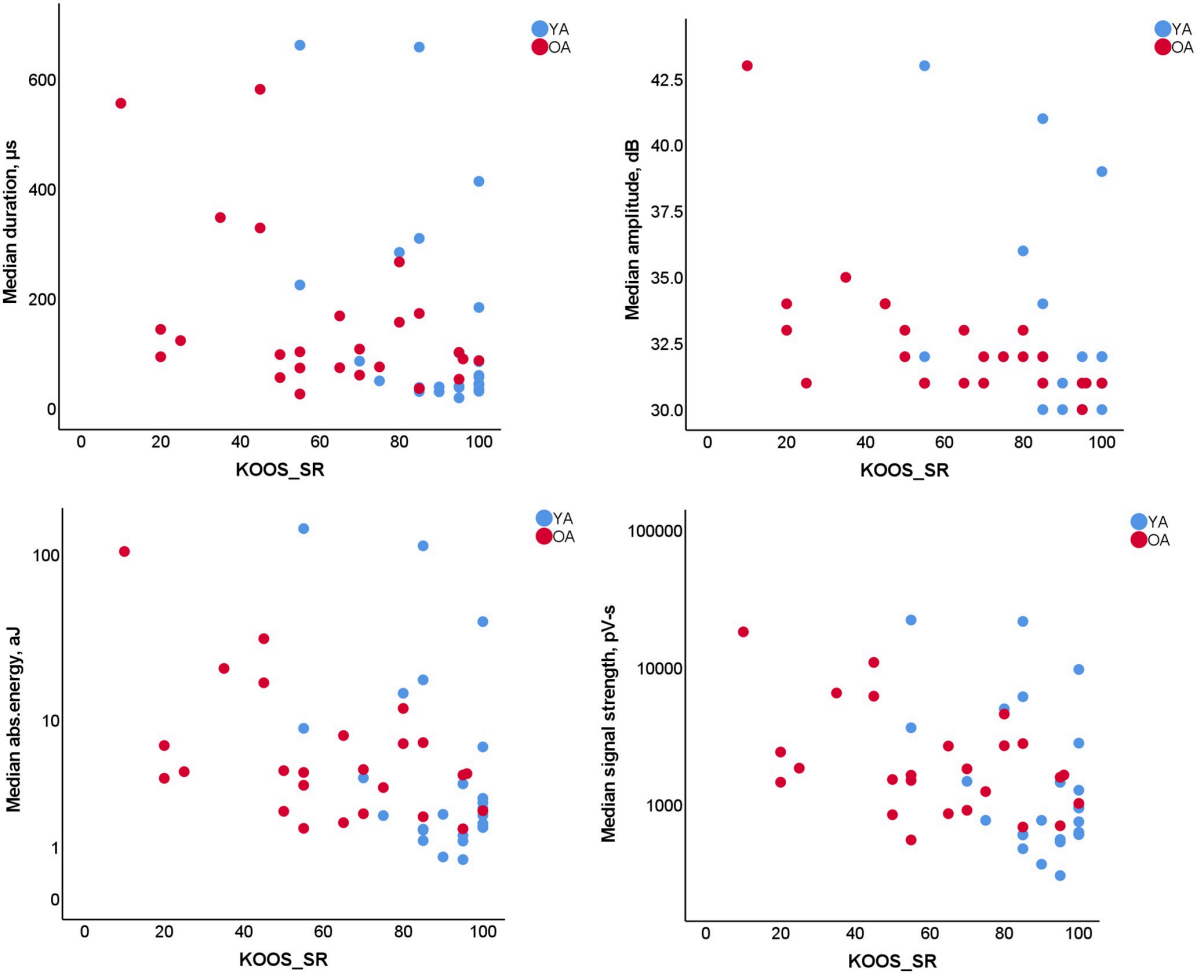
Scatter plots for moderate (Spearman’s ρ>0.4) correlations with KOOS_SR with lower frequency sensor, 60rpm cadence, 2^nd^ hit detection mode trial (LF_2_60).

The KOOS, KOOS_ADL and KOOS_SR subscales showed weak to moderate correlation with multiple knee AE parameters (Table 4, values in bold), particularly when using the LF sensor, a 60 rpm cadence, and hit detection modes 1 and 2 (LF_1/2_60). Overall, the KOOS_SR “Function in Sport and Recreation” subscale showed the highest correlation coefficients (Fig 2), with Spearman’s ρ above 0.4 for four AE parameters (median amplitude, duration, absolute energy, and signal strength). Correlation coefficients related to the KOOS_S “Other Symptoms” subscale were suggestive of a weak correlation only with the median amplitude (HF_1_30, LF_1/2_60) and absolute energy (LF_2_60). At the same time, the correlation with the answers to the subscale’s question regarding perceived noises from the knee (KOOS_S, question 2) returned even lower coefficient values, with only two types of trials (HF_1_30/60) showing an ρ above 0.3. Only one type of trial and a parameter (median amplitude, HF_1_30) were suggestive of a weak correlation with the KOOS_P “Pain” subscale.

**Table 4:** Spearman’s correlations between AE parameters and KOOS.

Trials	Median hit amplitude, dB			Median hit duration, μs			Median hit rise time, μs			Median hit absolute energy, attoJoules			Median hit signal strength, pV-s			Number of hits over 32 dB			Number of hits over 36dB		
	*ρ*	*CI (95%)*		*ρ*	*CI (95%)*		*ρ*	*CI (95%)*		*ρ*	*CI (95%)*		*ρ*	*CI (95%)*		*ρ*	*CI (95%)*		*ρ*	*CI (95%)*	
KOOS																					
***LF_1_30***	-.130	-.399	.159	-.112	-.383	.177	-.121	-.390	.169	-.171	-.433	.118	-.155	-.420	.134	-.030	-.310	.256	-.062	-.339	.226
***LF_2_30***	-.086	-.361	.202	-.077	-.352	.211	-.125	-.395	.164	-.080	-.355	.208	-.085	-.359	.204	-.024	-.305	.261	-.020	-.301	.265
***LF_3_30***	-.177	-.438	.112	-.129	-.398	.160	-.195	-.454	.093	-.194	-.453	.094	-.163	-.427	.126	-.132	-.400	.157	-.128	-.397	.161
***HF_1_30***	-.229	-.488	.068	-.058	-.345	.238	.058	-.238	.344	-.064	-.349	.232	-.048	-.335	.248	-.088	-.370	.210	-.056	-.342	.240
***HF_2_30***	-.067	-.355	.233	-.004	-.299	.292	-.010	-.304	.286	-.017	-.311	.279	-.014	-.308	.282	-.014	-.307	.283	-.012	-.306	.284
***HF_3_30***	.015	-.278	.306	.021	-.273	.311	-.053	-.340	.243	-.014	-.304	.280	-.007	-.298	.286	.101	-.197	.381	.120	-.178	.398
***LF_1_60***	**-.317**	-.553	-.033	**-.349**	-.577	-.070	**-.353**	-.581	-.075	**-.357**	-.584	-.079	**-.344**	-.574	-.064	-.190	-.452	.101	-.262	-.510	.026
***LF_2_60***	**-.410**	**-.623**	**-.141**	**-.344**	-.574	-.064	**-.283**	-.527	.003	**-.379**	-.600	-.104	**-.361**	-.587	-.084	-.135	-.405	.158	-.146	-.415	.146
***LF_3_60***	-.201	-.465	.097	-.246	-.502	.049	-.181	-.449	.117	**-.286**	-.534	.006	-.273	-.523	.021	.021	-.273	.311	.001	-.292	.293
***HF_1_60***	-.139	-.412	.156	-.011	-.299	.279	-.033	-.319	.258	-.016	-.303	.275	-.037	-.323	.255	-.068	-.351	.225	.003	-.286	.292
***HF_2_60***	-.073	-.354	.221	-.057	-.341	.236	-.012	-.300	.278	-.092	-.372	.202	-.050	-.334	.242	-.160	-.430	.135	-.091	-.371	.203
***HF_3_60***	-.249	-.500	.040	-.193	-.454	.098	-.134	-.405	.158	-.196	-.456	.096	-.170	-.435	.122	**-.317**	-.553	-.034	-.257	-.506	.032
**KOOS_ADL “Activities in Daily Life”**																					
***LF_1_30***	-.220	-.474	.067	-.186	-.446	.102	-.183	-.443	.106	-.246	-.495	.040	-.229	-.481	.058	-.115	-.386	.174	-.152	-.417	.137
***LF_2_30***	-.151	-.416	.138	-.133	-.401	.156	-.182	-.443	.107	-.144	-.411	.145	-.143	-.409	.147	-.121	-.391	.168	-.116	-.386	.173
***LF_3_30***	-.239	-.489	.048	-.221	-.475	.066	-.244	-.493	.043	-.264	-.509	.021	-.239	-.489	.047	-.208	-.464	.080	-.218	-.472	.069
***HF_1_30***	-.162	-.434	.137	-.069	-.354	.227	.019	-.275	.309	-.041	-.329	.255	-.048	-.336	.247	-.091	-.373	.207	-.056	-.342	.240
***HF_2_30***	-.029	-.321	.269	-.089	-.375	.211	-.088	-.373	.213	-.060	-.349	.239	-.065	-.353	.235	-.056	-.345	.244	-.045	-.336	.254
***HF_3_30***	.046	-.250	.333	-.026	-.315	.268	-.088	-.371	.209	-.036	-.325	.259	-.032	-.321	.263	.053	-.243	.340	.080	-.217	.364
***LF_1_60***	**-.322**	-.557	-.039	**-.346**	-.575	-.067	**-.366**	-.590	-.089	**-.375**	-.597	-.100	**-.364**	-.588	-.086	-.264	-.511	.024	**-.334**	-.566	-.053
***LF_2_60***	**-.403**	-.618	-.132	**-.363**	-.588	-.086	**-.283**	-.526	.004	**-.361**	-.587	-.084	**-.357**	-.584	-.079	-.203	-.463	.088	-.248	-.499	.041
***LF_3_60***	-.235	-.493	.061	**-.318**	-.558	-.028	-.230	-.489	.066	**-.335**	-.571	-.048	**-.326**	-.564	-.037	-.086	-.369	.211	-.122	-.400	.176
***HF_1_60***	-.089	-.369	.205	-.035	-.321	.256	-.058	-.341	.235	-.015	-.303	.275	-.030	-.316	.261	-.014	-.302	.276	.070	-.223	.352
***HF_2_60***	-.094	-.373	.201	-.128	-.402	.167	-.063	-.346	.230	-.141	-.413	.155	-.114	-.390	.181	-.147	-.419	.148	-.043	-.328	.249
***HF_3_60***	-.271	-.517	.017	-.240	-.493	.049	-.180	-.443	.112	-.272	-.518	.015	-.251	-.501	.038	**-.294**	-.535	-.008	-.251	-.501	.037
**KOOS_SR “Function in Sport and Recreation”**																					
***LF_1_30***	-.137	-.405	.152	-.131	-.400	.158	-.127	-.396	.162	-.200	-.457	.088	-.184	-.444	.105	-.077	-.352	.211	-.097	-.370	.192
***LF_2_30***	-.118	-.389	.171	-.107	-.379	.182	-.153	-.418	.137	-.114	-.384	.175	-.114	-.385	.175	-.077	-.353	.211	-.052	-.331	.234
***LF_3_30***	-.188	-.447	.101	-.136	-.404	.153	-.208	-.464	.080	-.204	-.461	.084	-.176	-.438	.113	-.151	-.417	.138	-.157	-.421	.133
***HF_1_30***	-.182	-.450	.117	-.092	-.374	.205	.026	-.268	.315	-.079	-.363	.218	-.081	-.364	.216	-.103	-.384	.195	-.073	-.358	.223
***HF_2_30***	-.049	-.339	.250	-.034	-.326	.264	-.031	-.323	.267	-.039	-.331	.259	-.048	-.338	.251	-.050	-.340	.249	-.060	-.349	.239
***HF_3_30***	.002	-.291	.294	-.030	-.320	.264	-.084	-.367	.213	-.067	-.352	.229	-.060	-.346	.236	.047	-.248	.335	.049	-.247	.336
***LF_1_60***	**-.320**	-.555	-.037	**-.369**	-.593	-.093	**-.376**	-.598	-.101	**-.381**	-.601	-.106	**-.366**	-.590	-.089	-.208	-.466	.084	**-.302**	-.541	-.017
***LF_2_60***	**-.441**	-.646	-.178	**-.409**	-.622	-.139	**-.332**	-.564	-.050	**-.427**	-.635	-.160	**-.414**	-.626	-.145	-.182	-.445	.110	-.233	-.487	.057
***LF_3_60***	-.251	-.506	.045	**-.329**	-.567	-.041	-.248	-.503	.048	**-.362**	-.591	-.078	**-.346**	-.579	-.060	-.022	-.312	.272	-.060	-.346	.236
***HF_1_60***	-.085	-.365	.209	-.065	-.347	.229	-.094	-.373	.200	-.054	-.338	.239	-.088	-.368	.206	-.071	-.353	.223	-.045	-.330	.247
***HF_2_60***	.014	-.276	.302	-.117	-.393	.178	-.089	-.369	.205	-.120	-.395	.175	-.097	-.375	.198	-.149	-.420	.147	-.145	-.417	.150
***HF_3_60***	-.188	-.450	.104	-.206	-.465	.085	-.163	-.429	.130	-.204	-.463	.088	-.182	-.445	.110	-.274	-.520	.013	-.253	-.503	.036

Color-coded values:p<0.005, p<0.01, p<0.05,p<0.1. Scatter plots of the examined variables, as well as *p*-values are included in the S3 Appendix.

### Functional assessment

**Fig 3:**
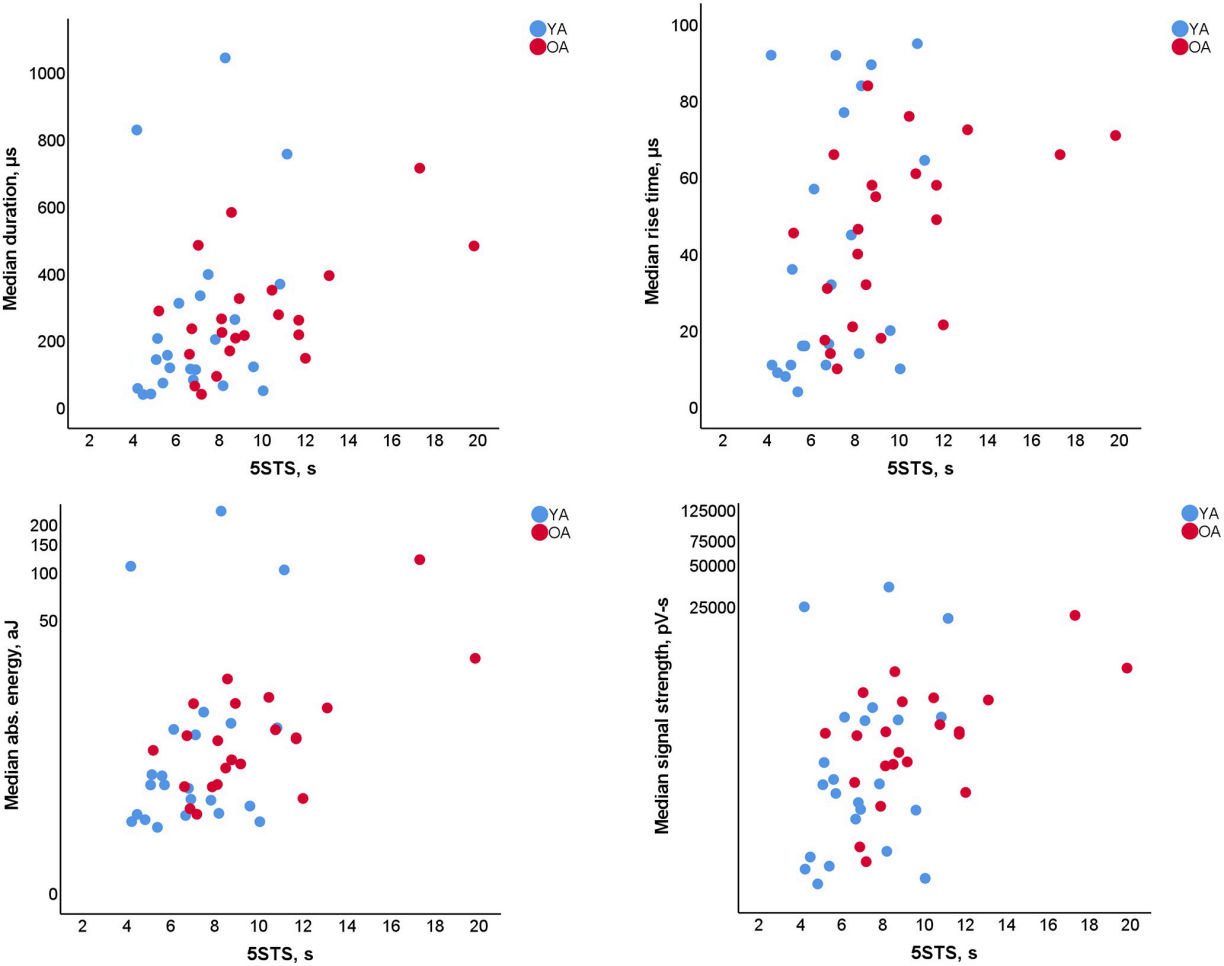
Scatter plots for moderate (Spearman’s ρ>0.4) correlations with 5STS time for trial with lower frequency sensor, 60rpm cadence, 3^rd^ hit detection mode trial (LF_3_60).

In most trials with 60 rpm cadence that were captured with the LF sensor (LF_1/2/3_60), the results were suggestive of a positive weak-to-moderate correlation for the median hit duration, rise time, absolute energy, and signal strength (Table 5, values in bold). Specifically, a moderate correlation was observed in the LF_3_60 trials with p-values less than 0.005, and Spearman’s ρ equal to 0.415 (0.129, 0.637) for the duration, 0.475 (0.202, 0.679) for the rise time, 0.435 (0.154, 0.651) for the absolute energy, and 0.433(0.151, 0.650) for the signal strength (Fig 3). Additionally, the same parameters were suggestive of a weak correlation in the 30 rpm cadence in combination with hit detection Mode 3 (LF_3_30). The HF_3_60 trials also suggest a weak correlation with the 5STS times, with a ρ above 0.3 for the same parameters.

**Table 5:** Spearman’s correlations between AE parameters and 5STS.

Trials	Median hit amplitude, dB			Median hit duration, μs			Median hit rise time, μs			Median hit absolute energy, attoJoules			Median hit signal strength, pV-s			Mean number of hits per rotation		
	ρ	*CI (95%)*		ρ	*CI (95%)*		ρ	*CI (95%)*		ρ	*CI (95%)*		ρ	*CI (95%)*		ρ	*CI (95%)*	
***LF_1_30***	.021	-.273	.311	.208	-.090	.471	.218	-.079	.480	.196	-.102	.462	.199	-.098	.464	-.067	-.353	.229
***LF_2_30***	.077	-.220	.361	.179	-.120	.447	**.302**	.011	.546	.165	-.134	.436	.194	-.104	.460	-.030	-.319	.265
***LF_3_30***	.236	-.060	.494	**.340**	.053	.575	**.382**	.102	.607	**.350**	.065	.583	**.352**	.067	.584	-.005	-.297	.287
***HF_1_30***	-.009	-.309	.294	.152	-.157	.434	.183	-.126	.459	.070	-.236	.364	.166	-.143	.446	**-.344**	-.585	-.047
***HF_2_30***	-.106	-.398	.206	.164	-.149	.446	.174	-.139	.455	.147	-.166	.432	.192	-.120	.470	-.270	-.532	.038
***HF_3_30***	-.085	-.377	.223	.193	-.116	.467	**.297**	-.005	.549	.219	-.088	.489	.219	-.088	.488	**-.354**	-.593	-.059
***LF_1_60***	.179	-.122	.451	**.305**	.010	.550	**.343**	.053	.579	**.311**	.018	.556	**.308**	.014	.553	-.080	-.367	.220
***LF_2_60***	.278	-.019	.530	**.414**	.136	.632	**.353**	.065	.587	**.356**	.068	.589	**.383**	.099	.609	.007	-.289	.301
***LF_3_60***	.161	-.148	.442	**.415**	.129	.637	**.475**	.202	.679	**.435**	.154	.651	**.433**	.151	.650	-.063	-.357	.244
***HF_1_60***	-.048	-.341	.254	.187	-.118	.459	.179	-.126	.453	.035	-.267	.330	.105	-.199	.391	-.172	-.448	.133
***HF_2_60***	-.106	-.392	.199	.289	-.010	.541	.227	-.076	.492	.169	-.136	.445	.215	-.089	.483	-.147	-.426	.159
***HF_3_60***	.083	-.217	.369	**.350**	.062	.585	**.399**	.117	.621	**.334**	.043	.573	**.352**	.064	.587	-.119	-.400	.183

Color-coded values:p<0.005, p<0.01, p<0.05,p<0.1. Scatter plots of the examined variables, as well as *p*-values are included in the in the S3 Appendix.

### Proprioception

No correlation with the TTDPM was found for most AE parameters (S3 Appendix), with only the number of hits per rotation, median hit amplitude and signal strength showing a weak correlation (ρ up to 0.361 (0.073, 0.593), p = 0.013) for the HF sensor with modes 2 and 3 (HF_2_60, HF_3_60).

### Age-related changes, BMI and motion smoothness

The relationship between age and changes in AE was previously explored by authors [54] for the same data. Only three parameters (AE event median duration, rise time, and signal strength) in two modes exhibited a weak correlation (p<0.05). Additionally, no significant correlations between BMI, log dimensionless jerk and AE parameters were observed (S3 Appendix).

## Discussion

### Self-reported assessment

The most commonly used criteria for osteoarthritis diagnosis are the width of the joint’s space [55], osteophytes [55] and cartilage thickness [56]. However, according to Illingworth et al. [7], joint space width was only weakly correlated with the perceived symptoms (KOOS_S) in the medial (*r* = 0.16) and lateral (*r* = 0.15) tibiofemoral spaces, while cartilage loss from MRIs was correlated with KOOS_S “Other Symptoms” (*r* = 0.36) and KOOS_P “Pain” (*r* = 0.34), but not with other KOOS subscales [7]. In contrast, joint AE parameters showed moderate correlation with all KOOS subscales (Table 4) apart from pain and symptoms. As expected, the highest correlation coefficients with joint AE parameters were observed for the subscales that involve assessments of the joints’ functional states (KOOS_SR and KOOS_ADL with ρ up to -0.441(-0.618, -0.132) and -0.403 (-0.618, -0.132), Table 4). These results confirm distinctive relationship between joint acoustics and its mechanical function, as altered AE events (e.g., signals with higher amplitude and longer duration) signify alterations in joint surfaces, such as cartilage wear or lubrication deficiency, which affect the knee’s ability to move smoothly and efficiently during functional tasks. Similarly, the lack of correlation with pain score indicates that AE events are largely determined by mechanical state of articular cartilage, which is not innervated, while pain is believed to be likely caused by secondary synovitis and bone marrow lesions [57].

This aspect of AE monitoring can potentially be beneficial in comprehensive assessment of treatments specifically aimed at improving mechanical function and mitigating the effect cartilage defects on joint movement, e.g., viscosupplementation. While pain and reported symptoms are subjective and can be influenced by various factors such as psychological state, individual pain perception, amount and nature of physical activity and other variables challenging to qualify, supplementing clinical trials with AE assessment of mechanical joint function can offer objective insights. This augmentation can aid clinicians to make more accurate evaluations of treatment efficiency as part of the complex assessment, which is particularly relevant in the context of clinical trials.

Interestingly, AE parameters showed either weak or no correlation with the "Other symptoms" KOOS subscale, and specifically with question 2, “Do you feel grinding, hear clicking, or any other type of noise when your knee moves?” (S3 Appendix). This suggests that a patient’s subjective perception of the knee’s audible noise might not be consistent with AE recordings in the considered frequency ranges, particularly in this instance where mostly mild knee complaints (median KOOS>70) were reported. Even though crepitus is considered a risk factor for the incidence of symptomatic knee osteoarthritis [58], in people with frequent knee symptoms and early signs of osteoarthritis (Kellgren and Lawrence grade 0–1) the presence of crepitus was not associated with structural osteoarthritis development over 4 years [59]. Joint assessments may benefit from objective sound-based tests, simultaneously reducing the influence of negative beliefs regarding crepitus [60] by distinguishing between non-pathological knee AE and acoustic events produced by damaged cartilage interaction during movement. However, while previous studies [16, 61] were able to differentiate osteoarthritic knees from healthy joint, further research, in particular regarding recording setup, is needed to establish exact features and thresholds of pathological joint AE events and bring the method to clinical practice.

### Functional assessment

In further support of our hypotheses, that AE events’ parameters correlated with functional assessment (with ρ up to 0.475 (0.202, 0.679), Table 5) of physical performance. Overall, AE parameters showed a larger association than the one previously reported by Kaukinen et al. for the femoral lateral cartilage degeneration assessed by MRI (effect size of 0.087 (0.001, 0.182)) [17]. While the exact relationship between cartilage state and poor physical function is still debated [17], the larger association between AE parameters and functional assessment compared to MRI findings implies that AE may be more sensitive in detecting early signs of joint disfunction. The AE monitoring provides an objective measure that allows joint assessment that considers how articulate surfaces interact during movement. Currently, videofluorography can be used to visualize internal structures of joints during movement; however, it comes with significant drawbacks of ionising radiation exposure and limited visualisation of soft tissues. While those issues do not apply to MRI, small operating volume makes it difficult to achieve the necessary range of motion in joints such as hip or knee. Moreover, taking into account the high costs and complexity of MRI and videofluorography, joint AE monitoring presents a potentially useful alternative in situations where it is difficult or impossible to use complex equipment, such as at-home treatment progress assessment, frequent check-ups or simple point of care assessment for osteoarthritis [62].

### Proprioception

In terms of proprioception, our results suggest only weak correlations (LF_1_30, HF_2/3_60, in the S3 Appendix) between proprioception testing using the TTPMD metric and AE parameters. While both methods were previously associated with joint articulate surface degeneration [14, 21], the complex nature of proprioception, the absence of an endorsed proprioception measurement technique [42], and the fact that our sample included mostly patients with mild knee complaints, may indicate that the correlation scores in this study might have been underestimated. The AE parameters that were indicative of possible weak correlation with the TTDPM did not match those correlated with the functional assessment test (Table 5), suggesting that the articular damage that produces pathological AE might differ in nature from the one causing a decline in proprioceptive ability. However, considering previous findings where no correlation was observed for majority of AE parameters and age contrary to results acquired in this study for functional assessment, it can be said that changes in knee AE are likely to be associated with pathological rather than physiological changes in cartilage structure [54].

### AE monitoring—Experimental setup

This study shown that BMI does not exhibit a significant correlation with AE parameters, while prior research has suggested a relationship between participants’ BMI and the recorded AE [36]; however, these findings were based on the sit-to-stand exercise, where joint load is directly influenced by the individuals’ weight. Simultaneously, excessive fat tissue surrounding the knee can have a dampening effect on recorded AE, potentially resulting in false negative results. In this study, the decision to exclude participants with higher BMI was made ensure soft tissue influence is minimised. However, higher BMI is associated with a higher risk of osteoarthritis, cartilage damage and decreased thickness [63, 64], and potentially excluding a significant portion of the population of interest cannot be considered practical. In future works, methods to account for fat tissue distribution and knee anatomy in general need to be developed. In previous studies authors suggested statistical and machine learning models [62] that account for BMI when diagnosing for osteoarthritis, however to the best of our knowledge models that take into account individual knee anatomy are yet to be developed.

Motion smoothness did not differ considerably between participants and was not correlated with AE parameters, together with that fact that most participants were able to keep requested cadence within 20% tolerance suggest that cycling offers better repeatability [34] in motion trajectory and speed and subsequently in produced knee AEs, than the exercise previously widely used in AE monitoring–knee flexion/extension injury [14]. While it can be suggested that participants with reported functional difficulties would potentially perform exercises less smoothly, resulting in increased AE due to motion artifacts, the results show that using cycling assuredly mitigates such issues. On the other hand, while cycling can be successfully used to monitor the knee joint, for joints with additional degrees of freedom, e.g., ball and socket joints, more complicated movements would need to be employed for a detailed investigation of the damaged articular surfaces.

The LF sensor performed better overall (Tables 4 and 5, values in bold), but correlation with 5STS times (up to 0.399 (0.117, 0.621) Spearman’s ρ for HF_3_60, Table 5) was also observed for the HF sensor, indicating that while a LF sensor might be the optimal choice, AE are spread across a wide range of information-bearing frequencies. However, it should also be noted that knee AEs were registered in a wide range of frequencies and using higher frequency potentially allows distinguishing smaller defects and micro damages; specifically, it has been reported that higher frequency AE hits are detected in osteoarthritic knees with thinner articular cartilage [16]. In that regard, recording signals with a high sampling frequency in a wide frequency range can help to achieve a more detailed picture of articular damage.

The setup of time parameters and threshold to isolate such events of interest is a wide research topic in non-destructive testing [18] but have not been considered in knee AE monitoring. While several studies previously identified a click-like event in post-processing [19, 20], those studies recorded knee using lower sampling rates (e.g., 100 kSps) than the ones presented in this study (5 MSps) and cannot be meaningfully compared. For the studies that employed similar frequency range, only default timing parameters were used to date [16].

The AE hit detection modes with shortened HDT and PDT values (mode 1 and 2, Table 3), showed higher correlation coefficients for most KOOS subscales (values in bold: Table 4), but not in the functional assessment (Table 5). A more advanced analysis of AE events, specifically, including the time component, might be beneficial in clinical applications. Moreover, our findings also encourage further investigations into the specifics of joint AE definition that, to the best of our knowledge, has not been done before, as using specific hit definition modes might be advantageous in diagnostics of certain conditions. The high sampling rate commonly used in AE monitoring allows to record AE signals with very high definition, but it also brings some limitations, as it is complicated to record and store full waveforms of continuous signals due to processing speed and memory constraints [65], especially in portable systems.

Within the context of performance measures, all trials with a higher cadence (LF_1/2/3_60, Table 5) showed higher coefficients than the lower cadence ones. This might be due to higher cadence recordings being more time consistent, than the more variable low cadence trials [34]. While median hit amplitude showed moderate correlation with KOOS, KOOS_SR, KOOS_ADL (Table 4), in the context of performance measures (Table 5) the median hit duration, rise time, absolute energy, and signal strength parameters showed moderate correlation scores with a p<0.05; thus, indicating that including the time component into an AE analysis may result in higher correlations with functional performance.

### Limitations and future work

To the best of the authors’ knowledge, this is the first study to investigate the association between knee AE and measures of functionality, patient satisfaction, and proprioception, specifically passive motion sensing. It has been shown that weak-to-moderate correlations exist for KOOS, timed 5STS, and knee AE parameters (Tables 4 and 5), while only a weak correlation was observed for the threshold of passive detection in proprioception testing (S3 Appendix). The sensor with a frequency range of 15–20 kHz provided better results when compared to higher frequency counterpart. AE events that were registered using hit definition parameters (Table 3) with shorter HDT and PDT than previously used in the literature [16, 24] returned higher correlation coefficients with the KOOS and its subscales (Table 4), but not 5STS time (Table 5). Overall, it can be said that using AE time parameters along with amplitude might be beneficial in further AE monitoring research. Additionally, the AE hit definition approaches in joint AE monitoring were not widely considered before, however the results of this study showed that the choice of hit definition mode is an important feature that should be investigated in future studies. The overview of key findings of this study, their potential implications and future work are summarised in the Table 6.

**Table 6:** Key findings.

Finding	Implication	Future work
AE parameters moderately correlate with KOOS and its subscales with correlation coefficients up to 0.441, p = 0.001.	Potential for the AE use in joint health assessment, specifically for at home, remote or continuous monitoring	Larger and diverse cohorts’ investigation: BMI range, joint conditions, age
Higher correlations with performance metric (5STS) up to 0.475, p = 0.001 and joint function subscales of KOOS (KOOS_SR, KOOS ADL).	AE likely indicates joint mechanical function, joint surface interaction	AE-aided mechanical joint state assessment, e.g., joint lubrication monitoring
		Investigation of underlying mechanisms of AE generation
No to poor correlation with pain and perceived (audible) knee noise/crepitus.	AEs likely reflect the non-innervated articular surfaces condition	Investigation of AE characteristics to differentiate pathological and non-pathological joint noise
	AE likely correlates with pathological knee sounds	
Higher correlations with lower frequency sensor recordings, amplitude/time and time-based AE parameters—median absolute energy, signal strength, rise time and duration, and high cadence cycling trials (60rpm).	Importance of recording setup: sensor frequency range, hit definition, movements used to excite AE	Further improvement of recording setup, extended cycling or assisted constrained movements investigation

While our findings are relevant to a wide range of AE monitoring applications for joint assessment, our measurements were obtained by sensors, processing and mounting techniques previously refined to reduce motion artefacts [33, 34] and might not be fully applicable to other experimental setups and frequency ranges. A further investigation using the full waveform of the AE signal might provide further insights. A wide range of ages was included to capture diverse knee conditions; however, the study cohort had a relatively small percentage of participants with KOOS below 70 and impaired proprioception. Considering this, higher correlation coefficients may be anticipated in future studies if participants with clinically proven grades of osteoarthritis and wider ranges of self-reported scores were to be tested.

While none of the participants reported osteoporosis, bone density was not clinically examined in the trial, which might have influenced the results for some patients and should be explored in future investigations. Additionally, while the sample was balanced between males and females, in the statistical evaluation we did not account for potential sex-specific differences. However, at least one study [13] previously reported that while AE was useful to provide complementary information to detect knee osteoarthritis in females, it was not the case for males. This, combined with the fact that females generally are at a higher risk of osteoarthritis [66] necessitates further investigation of sex-specific AE changes to enhance diagnostic value of the method. This study provides evidence of moderate correlations between AE parameters and a series of self-reported and function measures with a high degree of statistical certainty; however, weak or no associations should be interpreted with caution.

## Conclusion

Contrary to video fluoroscopy and other imaging technologies, joint AE monitoring presents a low-cost, portable, and relatively easily accessible technology that has the potential to become a complementary modality to imaging methods currently used in clinical practice as it provides an objective insight into the interaction between cartilage surfaces during knee movement. This is particularly useful since both MRI and radiography is mostly correlated with pain and osteoarthritis-related symptoms, rather than the functional assessments of the joint. Considering the correlation coefficients (up to 0.475 (0.202, 0.679), p = 0.001, Tables 4 and 5) with both functional measures and patient reported measures, AE monitoring can be useful in quantifying the effects of drugs and therapies that influence interaction between articular surfaces, such as hyaluronic injections, and during remote monitoring and at-home rehabilitation, where the use of imaging technologies is limited.

## Supporting information

S1 Appendix Descriptive statistics AE parameters. (DOCX)

S2 Appendix Anthropometrics, KOOS, 5STS, TTDPM. (DOCX)

S3 Appendix Spearman’s correlations. (XLSX)
