# Supplementary material for: Correlation between proprioception, functionality, patient-reported knee condition and joint acoustic emissions
Source: PLoS One. 2024 Nov 6;19(11):e0310123. doi: 10.1371/journal.pone.0310123 (PMC11540232; doi:10.1371/journal.pone.0310123)
Supplement: S1 Appendix — (DOCX) [file pone.0310123.s001.docx]

# Notations

*Sensor_Mode_Cadence* notation used for each type of trial, referring to the low (LF) or high frequency (HF) Sensors, the Modes of hit detection (1/2/3, Table 3 of the manuscript), and 60 or 30 rpm Cadences. LF_3_60, for example, denoted a trial with the PK31 (LF) sensor, mode 3, at 60 rpm.

| **Summary** | | | | | | |
| --- | --- | --- | --- | --- | --- | --- |
|  | Cases | | | | | |
|  | Valid | | Missing | | Total | |
|  | N | Percent | N | Percent | N | Percent |
| LF_1_30 | 51 | 100.0% | 0 | 0.0% | 51 | 100.0% |
| LF_2_30 | 51 | 100.0% | 0 | 0.0% | 51 | 100.0% |
| LF_3_30 | 51 | 100.0% | 0 | 0.0% | 51 | 100.0% |
| HF_1_30 | 48 | 94.1% | 3 | 5.9% | 51 | 100.0% |
| HF_2_30 | 47 | 92.2% | 4 | 7.8% | 51 | 100.0% |
| HF_3_30 | 48 | 94.1% | 3 | 5.9% | 51 | 100.0% |
| LF_1_60 | 50 | 98.0% | 1 | 2.0% | 51 | 100.0% |
| LF_2_60 | 50 | 98.0% | 1 | 2.0% | 51 | 100.0% |
| LF_3_60 | 48 | 94.1% | 3 | 5.9% | 51 | 100.0% |
| HF_1_60 | 49 | 96.1% | 2 | 3.9% | 51 | 100.0% |
| HF_2_60 | 49 | 96.1% | 2 | 3.9% | 51 | 100.0% |
| HF_3_60 | 50 | 98.0% | 1 | 2.0% | 51 | 100.0% |

| **Descriptive statistics: Median Hit Amplitude, dB** | | | |
| --- | --- | --- | --- |
|  | | Statistic | Std. Error |
| LF_1_30 | Mean | 33.588 | 0.663 |
|  | Median | 32.000 |  |
|  | Variance | 22.397 |  |
|  | Std. Deviation | 4.733 |  |
|  | Minimum | 29.000 |  |
|  | Maximum | 52.000 |  |
|  | Range | 23.000 |  |
|  | Interquartile Range | 4.000 |  |
| LF_2_30 | Mean | 34.422 | 0.647 |
|  | Median | 32.500 |  |
|  | Variance | 21.334 |  |
|  | Std. Deviation | 4.619 |  |
|  | Minimum | 30.000 |  |
|  | Maximum | 49.000 |  |
|  | Range | 19.000 |  |
|  | Interquartile Range | 5.000 |  |
| LF_3_30 | Mean | 34.120 | 0.601 |
|  | Median | 33.000 |  |
|  | Variance | 18.426 |  |
|  | Std. Deviation | 4.293 |  |
|  | Minimum | 30.000 |  |
|  | Maximum | 54.000 |  |
|  | Range | 24.000 |  |
|  | Interquartile Range | 5.000 |  |
| HF_1_30 | Mean | 24.563 | 0.114 |
|  | Median | 24.000 |  |
|  | Variance | 0.624 |  |
|  | Std. Deviation | 0.790 |  |
|  | Minimum | 23.500 |  |
|  | Maximum | 27.000 |  |
|  | Range | 3.500 |  |
|  | Interquartile Range | 1.000 |  |
| HF_2_30 | Mean | 24.532 | 0.100 |
|  | Median | 24.000 |  |
|  | Variance | 0.472 |  |
|  | Std. Deviation | 0.687 |  |
|  | Minimum | 24.000 |  |
|  | Maximum | 26.000 |  |
|  | Range | 2.000 |  |
|  | Interquartile Range | 1.000 |  |
| HF_3_30 | Mean | 24.656 | 0.122 |
|  | Median | 24.000 |  |
|  | Variance | 0.714 |  |
|  | Std. Deviation | 0.845 |  |
|  | Minimum | 24.000 |  |
|  | Maximum | 27.000 |  |
|  | Range | 3.000 |  |
|  | Interquartile Range | 1.000 |  |
| LF_1_60 | Mean | 32.310 | 0.413 |
|  | Median | 32.000 |  |
|  | Variance | 8.509 |  |
|  | Std. Deviation | 2.917 |  |
|  | Minimum | 29.000 |  |
|  | Maximum | 43.000 |  |
|  | Range | 14.000 |  |
|  | Interquartile Range | 2.000 |  |
| LF_2_60 | Mean | 32.540 | 0.426 |
|  | Median | 32.000 |  |
|  | Variance | 9.070 |  |
|  | Std. Deviation | 3.012 |  |
|  | Minimum | 30.000 |  |
|  | Maximum | 43.000 |  |
|  | Range | 13.000 |  |
|  | Interquartile Range | 2.000 |  |
| LF_3_60 | Mean | 32.719 | 0.448 |
|  | Median | 32.000 |  |
|  | Variance | 9.627 |  |
|  | Std. Deviation | 3.103 |  |
|  | Minimum | 30.000 |  |
|  | Maximum | 44.000 |  |
|  | Range | 14.000 |  |
|  | Interquartile Range | 2.000 |  |
| HF_1_60 | Mean | 25.000 | 0.154 |
|  | Median | 25.000 |  |
|  | Variance | 1.167 |  |
|  | Std. Deviation | 1.080 |  |
|  | Minimum | 24.000 |  |
|  | Maximum | 29.000 |  |
|  | Range | 5.000 |  |
|  | Interquartile Range | 1.000 |  |
| HF_2_60 | Mean | 24.888 | 0.130 |
|  | Median | 25.000 |  |
|  | Variance | 0.826 |  |
|  | Std. Deviation | 0.909 |  |
|  | Minimum | 24.000 |  |
|  | Maximum | 28.000 |  |
|  | Range | 4.000 |  |
|  | Interquartile Range | 1.000 |  |
| HF_3_60 | Mean | 24.880 | 0.130 |
|  | Median | 25.000 |  |
|  | Variance | 0.842 |  |
|  | Std. Deviation | 0.918 |  |
|  | Minimum | 24.000 |  |
|  | Maximum | 28.000 |  |
|  | Range | 4.000 |  |
|  | Interquartile Range | 1.000 |  |

| **Tests of Normality: Median Hit Amplitude, dB** | | | |
| --- | --- | --- | --- |
|  | Shapiro-Wilk | | |
|  | Statistic | df | Sig. |
| LF_1_30 | .706 | 51 | <.001 |
| LF_2_30 | .766 | 51 | <.001 |
| LF_3_30 | .761 | 51 | <.001 |
| HF_1_30 | .768 | 48 | <.001 |
| HF_2_30 | .719 | 47 | <.001 |
| HF_3_30 | .755 | 48 | <.001 |
| LF_1_60 | .722 | 50 | <.001 |
| LF_2_60 | .677 | 50 | <.001 |
| LF_3_60 | .687 | 48 | <.001 |
| HF_1_60 | .799 | 49 | <.001 |
| HF_2_60 | .819 | 49 | <.001 |
| HF_3_60 | .800 | 50 | <.001 |

| **Descriptive statistics: Median Hit Duration, µs** | | | |
| --- | --- | --- | --- |
|  | | Statistic | Std. Error |
| LF_1_30 | Mean | 141.147 | 32.123 |
|  | Median | 49.500 |  |
|  | Variance | 52625.623 |  |
|  | Std. Deviation | 229.403 |  |
|  | Minimum | 6.000 |  |
|  | Maximum | 896.500 |  |
|  | Range | 890.500 |  |
|  | Interquartile Range | 66.000 |  |
| LF_2_30 | Mean | 262.412 | 39.400 |
|  | Median | 145.000 |  |
|  | Variance | 79171.317 |  |
|  | Std. Deviation | 281.374 |  |
|  | Minimum | 6.000 |  |
|  | Maximum | 1331.500 |  |
|  | Range | 1325.500 |  |
|  | Interquartile Range | 243.500 |  |
| LF_3_30 | Mean | 364.324 | 46.500 |
|  | Median | 274.000 |  |
|  | Variance | 110273.718 |  |
|  | Std. Deviation | 332.075 |  |
|  | Minimum | 46.500 |  |
|  | Maximum | 2120.000 |  |
|  | Range | 2073.500 |  |
|  | Interquartile Range | 352.500 |  |
| HF_1_30 | Mean | 53.260 | 11.093 |
|  | Median | 28.000 |  |
|  | Variance | 5906.755 |  |
|  | Std. Deviation | 76.855 |  |
|  | Minimum | 19.000 |  |
|  | Maximum | 409.000 |  |
|  | Range | 390.000 |  |
|  | Interquartile Range | 9.900 |  |
| HF_2_30 | Mean | 61.277 | 8.980 |
|  | Median | 41.000 |  |
|  | Variance | 3790.096 |  |
|  | Std. Deviation | 61.564 |  |
|  | Minimum | 20.000 |  |
|  | Maximum | 321.000 |  |
|  | Range | 301.000 |  |
|  | Interquartile Range | 32.000 |  |
| HF_3_30 | Mean | 138.958 | 18.098 |
|  | Median | 73.000 |  |
|  | Variance | 15721.222 |  |
|  | Std. Deviation | 125.384 |  |
|  | Minimum | 29.000 |  |
|  | Maximum | 648.000 |  |
|  | Range | 619.000 |  |
|  | Interquartile Range | 154.100 |  |
| LF_1_60 | Mean | 77.270 | 15.824 |
|  | Median | 37.000 |  |
|  | Variance | 12519.931 |  |
|  | Std. Deviation | 111.893 |  |
|  | Minimum | 6.000 |  |
|  | Maximum | 497.000 |  |
|  | Range | 491.000 |  |
|  | Interquartile Range | 51.000 |  |
| LF_2_60 | Mean | 151.750 | 23.625 |
|  | Median | 86.500 |  |
|  | Variance | 27905.849 |  |
|  | Std. Deviation | 167.050 |  |
|  | Minimum | 19.000 |  |
|  | Maximum | 662.500 |  |
|  | Range | 643.500 |  |
|  | Interquartile Range | 133.400 |  |
| LF_3_60 | Mean | 262.896 | 31.526 |
|  | Median | 207.500 |  |
|  | Variance | 47705.521 |  |
|  | Std. Deviation | 218.416 |  |
|  | Minimum | 40.000 |  |
|  | Maximum | 1044.000 |  |
|  | Range | 1004.000 |  |
|  | Interquartile Range | 215.500 |  |
| HF_1_60 | Mean | 50.163 | 10.752 |
|  | Median | 27.000 |  |
|  | Variance | 5664.160 |  |
|  | Std. Deviation | 75.261 |  |
|  | Minimum | 19.000 |  |
|  | Maximum | 385.000 |  |
|  | Range | 366.000 |  |
|  | Interquartile Range | 12.500 |  |
| HF_2_60 | Mean | 67.531 | 10.818 |
|  | Median | 44.000 |  |
|  | Variance | 5734.338 |  |
|  | Std. Deviation | 75.725 |  |
|  | Minimum | 22.000 |  |
|  | Maximum | 379.000 |  |
|  | Range | 357.000 |  |
|  | Interquartile Range | 21.500 |  |
| HF_3_60 | Mean | 146.390 | 18.871 |
|  | Median | 98.500 |  |
|  | Variance | 17806.483 |  |
|  | Std. Deviation | 133.441 |  |
|  | Minimum | 31.500 |  |
|  | Maximum | 683.500 |  |
|  | Range | 652.000 |  |
|  | Interquartile Range | 137.500 |  |

| **Tests of Normality: Median Hit Duration, µs** | | | |
| --- | --- | --- | --- |
|  | Shapiro-Wilk | | |
|  | Statistic | df | Sig. |
| LF_1_30 | .568 | 51 | <.001 |
| LF_2_30 | .785 | 51 | <.001 |
| LF_3_30 | .715 | 51 | <.001 |
| HF_1_30 | .452 | 48 | <.001 |
| HF_2_30 | .554 | 47 | <.001 |
| HF_3_30 | .764 | 48 | <.001 |
| LF_1_60 | .591 | 50 | <.001 |
| LF_2_60 | .720 | 50 | <.001 |
| LF_3_60 | .824 | 48 | <.001 |
| HF_1_60 | .414 | 49 | <.001 |
| HF_2_60 | .493 | 49 | <.001 |
| HF_3_60 | .730 | 50 | <.001 |

| **Descriptive statistics: Median Rise Time, µs** | | | |
| --- | --- | --- | --- |
|  | | Statistic | Std. Error |
| LF_1_30 | Mean | 30.108 | 4.286 |
|  | Median | 17.000 |  |
|  | Variance | 936.683 |  |
|  | Std. Deviation | 30.605 |  |
|  | Minimum | 1.000 |  |
|  | Maximum | 149.000 |  |
|  | Range | 148.000 |  |
|  | Interquartile Range | 29.000 |  |
| LF_2_30 | Mean | 44.951 | 5.997 |
|  | Median | 34.000 |  |
|  | Variance | 1833.973 |  |
|  | Std. Deviation | 42.825 |  |
|  | Minimum | 2.000 |  |
|  | Maximum | 246.500 |  |
|  | Range | 244.500 |  |
|  | Interquartile Range | 42.000 |  |
| LF_3_30 | Mean | 54.167 | 4.553 |
|  | Median | 54.000 |  |
|  | Variance | 1057.387 |  |
|  | Std. Deviation | 32.518 |  |
|  | Minimum | 8.000 |  |
|  | Maximum | 155.000 |  |
|  | Range | 147.000 |  |
|  | Interquartile Range | 38.500 |  |
| HF_1_30 | Mean | 22.958 | 2.831 |
|  | Median | 17.500 |  |
|  | Variance | 384.573 |  |
|  | Std. Deviation | 19.611 |  |
|  | Minimum | 5.000 |  |
|  | Maximum | 87.500 |  |
|  | Range | 82.500 |  |
|  | Interquartile Range | 13.000 |  |
| HF_2_30 | Mean | 29.851 | 2.669 |
|  | Median | 27.000 |  |
|  | Variance | 334.673 |  |
|  | Std. Deviation | 18.294 |  |
|  | Minimum | 5.000 |  |
|  | Maximum | 99.000 |  |
|  | Range | 94.000 |  |
|  | Interquartile Range | 20.000 |  |
| HF_3_30 | Mean | 44.948 | 3.782 |
|  | Median | 38.000 |  |
|  | Variance | 686.683 |  |
|  | Std. Deviation | 26.205 |  |
|  | Minimum | 6.000 |  |
|  | Maximum | 107.000 |  |
|  | Range | 101.000 |  |
|  | Interquartile Range | 38.800 |  |
| LF_1_60 | Mean | 20.180 | 2.742 |
|  | Median | 11.000 |  |
|  | Variance | 375.865 |  |
|  | Std. Deviation | 19.387 |  |
|  | Minimum | 2.000 |  |
|  | Maximum | 73.000 |  |
|  | Range | 71.000 |  |
|  | Interquartile Range | 26.500 |  |
| LF_2_60 | Mean | 32.350 | 3.853 |
|  | Median | 29.500 |  |
|  | Variance | 742.145 |  |
|  | Std. Deviation | 27.242 |  |
|  | Minimum | 2.000 |  |
|  | Maximum | 113.000 |  |
|  | Range | 111.000 |  |
|  | Interquartile Range | 42.800 |  |
| LF_3_60 | Mean | 42.146 | 4.001 |
|  | Median | 37.000 |  |
|  | Variance | 768.468 |  |
|  | Std. Deviation | 27.721 |  |
|  | Minimum | 4.000 |  |
|  | Maximum | 95.000 |  |
|  | Range | 91.000 |  |
|  | Interquartile Range | 49.500 |  |
| HF_1_60 | Mean | 20.582 | 2.410 |
|  | Median | 15.500 |  |
|  | Variance | 284.629 |  |
|  | Std. Deviation | 16.871 |  |
|  | Minimum | 5.000 |  |
|  | Maximum | 93.000 |  |
|  | Range | 88.000 |  |
|  | Interquartile Range | 8.300 |  |
| HF_2_60 | Mean | 28.071 | 2.666 |
|  | Median | 21.000 |  |
|  | Variance | 348.198 |  |
|  | Std. Deviation | 18.660 |  |
|  | Minimum | 7.000 |  |
|  | Maximum | 78.000 |  |
|  | Range | 71.000 |  |
|  | Interquartile Range | 16.300 |  |
| HF_3_60 | Mean | 48.670 | 6.913 |
|  | Median | 37.500 |  |
|  | Variance | 2389.516 |  |
|  | Std. Deviation | 48.883 |  |
|  | Minimum | 7.000 |  |
|  | Maximum | 346.500 |  |
|  | Range | 339.500 |  |
|  | Interquartile Range | 34.500 |  |

| **Tests of Normality: Median Rise Time, µs** | | | |
| --- | --- | --- | --- |
|  | Shapiro-Wilk | | |
|  | Statistic | df | Sig. |
| LF_1_30 | .801 | 51 | <.001 |
| LF_2_30 | .787 | 51 | <.001 |
| LF_3_30 | .940 | 51 | .012 |
| HF_1_30 | .716 | 48 | <.001 |
| HF_2_30 | .866 | 47 | <.001 |
| HF_3_30 | .930 | 48 | .007 |
| LF_1_60 | .821 | 50 | <.001 |
| LF_2_60 | .876 | 50 | <.001 |
| LF_3_60 | .921 | 48 | .003 |
| HF_1_60 | .665 | 49 | <.001 |
| HF_2_60 | .805 | 49 | <.001 |
| HF_3_60 | .536 | 50 | <.001 |

| **Descriptive statistics: Median Absolute Energy, attoJoules** | | | |
| --- | --- | --- | --- |
|  | | Statistic | Std. Error |
| LF_1_30 | Mean | 60.801 | 32.138 |
|  | Median | 3.066 |  |
|  | Variance | 52674.512 |  |
|  | Std. Deviation | 229.509 |  |
|  | Minimum | 0.369 |  |
|  | Maximum | 1468.500 |  |
|  | Range | 1468.131 |  |
|  | Interquartile Range | 5.496 |  |
| LF_2_30 | Mean | 51.881 | 20.130 |
|  | Median | 5.665 |  |
|  | Variance | 20666.869 |  |
|  | Std. Deviation | 143.760 |  |
|  | Minimum | 0.487 |  |
|  | Maximum | 835.558 |  |
|  | Range | 835.070 |  |
|  | Interquartile Range | 16.076 |  |
| LF_3_30 | Mean | 75.381 | 52.131 |
|  | Median | 9.195 |  |
|  | Variance | 138597.286 |  |
|  | Std. Deviation | 372.287 |  |
|  | Minimum | 1.710 |  |
|  | Maximum | 2671.000 |  |
|  | Range | 2669.290 |  |
|  | Interquartile Range | 23.728 |  |
| HF_1_30 | Mean | 0.688 | 0.215 |
|  | Median | 0.271 |  |
|  | Variance | 2.214 |  |
|  | Std. Deviation | 1.488 |  |
|  | Minimum | 0.166 |  |
|  | Maximum | 7.507 |  |
|  | Range | 7.341 |  |
|  | Interquartile Range | 0.117 |  |
| HF_2_30 | Mean | 0.585 | 0.120 |
|  | Median | 0.375 |  |
|  | Variance | 0.678 |  |
|  | Std. Deviation | 0.824 |  |
|  | Minimum | 0.207 |  |
|  | Maximum | 5.243 |  |
|  | Range | 5.036 |  |
|  | Interquartile Range | 0.216 |  |
| HF_3_30 | Mean | 1.054 | 0.233 |
|  | Median | 0.609 |  |
|  | Variance | 2.600 |  |
|  | Std. Deviation | 1.612 |  |
|  | Minimum | 0.362 |  |
|  | Maximum | 10.799 |  |
|  | Range | 10.437 |  |
|  | Interquartile Range | 0.523 |  |
| LF_1_60 | Mean | 9.483 | 3.319 |
|  | Median | 1.872 |  |
|  | Variance | 550.908 |  |
|  | Std. Deviation | 23.471 |  |
|  | Minimum | 0.410 |  |
|  | Maximum | 123.941 |  |
|  | Range | 123.531 |  |
|  | Interquartile Range | 3.832 |  |
| LF_2_60 | Mean | 12.883 | 4.044 |
|  | Median | 3.631 |  |
|  | Variance | 817.600 |  |
|  | Std. Deviation | 28.594 |  |
|  | Minimum | 0.694 |  |
|  | Maximum | 142.068 |  |
|  | Range | 141.374 |  |
|  | Interquartile Range | 5.555 |  |
| LF_3_60 | Mean | 18.340 | 6.099 |
|  | Median | 5.577 |  |
|  | Variance | 1785.635 |  |
|  | Std. Deviation | 42.257 |  |
|  | Minimum | 1.589 |  |
|  | Maximum | 243.542 |  |
|  | Range | 241.953 |  |
|  | Interquartile Range | 7.488 |  |
| HF_1_60 | Mean | 0.703 | 0.237 |
|  | Median | 0.257 |  |
|  | Variance | 2.743 |  |
|  | Std. Deviation | 1.656 |  |
|  | Minimum | 0.155 |  |
|  | Maximum | 8.585 |  |
|  | Range | 8.430 |  |
|  | Interquartile Range | 0.166 |  |
| HF_2_60 | Mean | 0.653 | 0.150 |
|  | Median | 0.354 |  |
|  | Variance | 1.103 |  |
|  | Std. Deviation | 1.050 |  |
|  | Minimum | 0.219 |  |
|  | Maximum | 5.524 |  |
|  | Range | 5.305 |  |
|  | Interquartile Range | 0.225 |  |
| HF_3_60 | Mean | 0.931 | 0.165 |
|  | Median | 0.637 |  |
|  | Variance | 1.362 |  |
|  | Std. Deviation | 1.167 |  |
|  | Minimum | 0.345 |  |
|  | Maximum | 7.035 |  |
|  | Range | 6.690 |  |
|  | Interquartile Range | 0.313 |  |

| **Tests of Normality: Median Absolute Energy, attoJoules** | | | |
| --- | --- | --- | --- |
|  | Shapiro-Wilk | | |
|  | Statistic | df | Sig. |
| LF_1_30 | .283 | 51 | <.001 |
| LF_2_30 | .386 | 51 | <.001 |
| LF_3_30 | .173 | 51 | <.001 |
| HF_1_30 | .354 | 48 | <.001 |
| HF_2_30 | .416 | 47 | <.001 |
| HF_3_30 | .389 | 48 | <.001 |
| LF_1_60 | .407 | 50 | <.001 |
| LF_2_60 | .435 | 50 | <.001 |
| LF_3_60 | .404 | 48 | <.001 |
| HF_1_60 | .336 | 49 | <.001 |
| HF_2_60 | .397 | 49 | <.001 |
| HF_3_60 | .428 | 50 | <.001 |

| **Descriptive statistics: Median signal strength, pV-s** | | | |
| --- | --- | --- | --- |
|  | | Statistic | Std. Error |
| LF_1_30 | Mean | 5947.804 | 2051.531 |
|  | Median | 1058.000 |  |
|  | Variance | 214647701.105 |  |
|  | Std. Deviation | 14650.860 |  |
|  | Minimum | 131.150 |  |
|  | Maximum | 78328.500 |  |
|  | Range | 78197.350 |  |
|  | Interquartile Range | 1693.925 |  |
| LF_2_30 | Mean | 7714.972 | 1964.103 |
|  | Median | 2242.000 |  |
|  | Variance | 196742815.361 |  |
|  | Std. Deviation | 14026.504 |  |
|  | Minimum | 158.600 |  |
|  | Maximum | 74638.000 |  |
|  | Range | 74479.400 |  |
|  | Interquartile Range | 5124.000 |  |
| LF_3_30 | Mean | 9170.084 | 2946.784 |
|  | Median | 3796.000 |  |
|  | Variance | 442860382.066 |  |
|  | Std. Deviation | 21044.248 |  |
|  | Minimum | 765.550 |  |
|  | Maximum | 149057.000 |  |
|  | Range | 148291.450 |  |
|  | Interquartile Range | 7564.000 |  |
| HF_1_30 | Mean | 428.241 | 106.768 |
|  | Median | 224.175 |  |
|  | Variance | 547167.614 |  |
|  | Std. Deviation | 739.708 |  |
|  | Minimum | 135.725 |  |
|  | Maximum | 3852.000 |  |
|  | Range | 3716.275 |  |
|  | Interquartile Range | 79.300 |  |
| HF_2_30 | Mean | 446.690 | 78.270 |
|  | Median | 318.725 |  |
|  | Variance | 287932.677 |  |
|  | Std. Deviation | 536.594 |  |
|  | Minimum | 149.450 |  |
|  | Maximum | 3392.000 |  |
|  | Range | 3242.550 |  |
|  | Interquartile Range | 201.300 |  |
| HF_3_30 | Mean | 816.038 | 128.485 |
|  | Median | 495.625 |  |
|  | Variance | 792397.875 |  |
|  | Std. Deviation | 890.167 |  |
|  | Minimum | 237.900 |  |
|  | Maximum | 5694.000 |  |
|  | Range | 5456.100 |  |
|  | Interquartile Range | 539.469 |  |
| LF_1_60 | Mean | 2105.195 | 579.406 |
|  | Median | 674.050 |  |
|  | Variance | 16785593.086 |  |
|  | Std. Deviation | 4097.022 |  |
|  | Minimum | 149.450 |  |
|  | Maximum | 20054.000 |  |
|  | Range | 19904.550 |  |
|  | Interquartile Range | 1100.025 |  |
| LF_2_60 | Mean | 3239.465 | 711.328 |
|  | Median | 1461.750 |  |
|  | Variance | 25299369.429 |  |
|  | Std. Deviation | 5029.848 |  |
|  | Minimum | 305.000 |  |
|  | Maximum | 22218.000 |  |
|  | Range | 21913.000 |  |
|  | Interquartile Range | 2101.513 |  |
| LF_3_60 | Mean | 4756.178 | 984.183 |
|  | Median | 2293.500 |  |
|  | Variance | 46493576.239 |  |
|  | Std. Deviation | 6818.620 |  |
|  | Minimum | 730.475 |  |
|  | Maximum | 34692.000 |  |
|  | Range | 33961.525 |  |
|  | Interquartile Range | 2965.500 |  |
| HF_1_60 | Mean | 426.597 | 116.416 |
|  | Median | 192.150 |  |
|  | Variance | 664082.160 |  |
|  | Std. Deviation | 814.912 |  |
|  | Minimum | 118.950 |  |
|  | Maximum | 4081.000 |  |
|  | Range | 3962.050 |  |
|  | Interquartile Range | 89.975 |  |
| HF_2_60 | Mean | 483.427 | 92.025 |
|  | Median | 282.125 |  |
|  | Variance | 414965.736 |  |
|  | Std. Deviation | 644.178 |  |
|  | Minimum | 158.600 |  |
|  | Maximum | 3190.000 |  |
|  | Range | 3031.400 |  |
|  | Interquartile Range | 186.813 |  |
| HF_3_60 | Mean | 762.654 | 102.415 |
|  | Median | 538.325 |  |
|  | Variance | 524446.546 |  |
|  | Std. Deviation | 724.187 |  |
|  | Minimum | 244.000 |  |
|  | Maximum | 4182.000 |  |
|  | Range | 3938.000 |  |
|  | Interquartile Range | 354.181 |  |

| **Tests of Normality: Median signal strength, pV-s** | | | |
| --- | --- | --- | --- |
|  | Shapiro-Wilk | | |
|  | Statistic | df | Sig. |
| LF_1_30 | .426 | 51 | <.001 |
| LF_2_30 | .538 | 51 | <.001 |
| LF_3_30 | .339 | 51 | <.001 |
| HF_1_30 | .396 | 48 | <.001 |
| HF_2_30 | .467 | 47 | <.001 |
| HF_3_30 | .552 | 48 | <.001 |
| LF_1_60 | .477 | 50 | <.001 |
| LF_2_60 | .569 | 50 | <.001 |
| LF_3_60 | .564 | 48 | <.001 |
| HF_1_60 | .368 | 49 | <.001 |
| HF_2_60 | .438 | 49 | <.001 |
| HF_3_60 | .571 | 50 | <.001 |

| **Descriptive statistics: Number of hits over 32dB** | | | |
| --- | --- | --- | --- |
|  | | Statistic | Std. Error |
| LF_1_30 | Mean | 117.980 | 11.922 |
|  | Median | 98.000 |  |
|  | Variance | 7249.020 |  |
|  | Std. Deviation | 85.141 |  |
|  | Minimum | 6.000 |  |
|  | Maximum | 359.000 |  |
|  | Range | 353.000 |  |
|  | Interquartile Range | 124.000 |  |
| LF_2_30 | Mean | 139.610 | 14.294 |
|  | Median | 107.000 |  |
|  | Variance | 10420.163 |  |
|  | Std. Deviation | 102.079 |  |
|  | Minimum | 6.000 |  |
|  | Maximum | 435.000 |  |
|  | Range | 429.000 |  |
|  | Interquartile Range | 150.000 |  |
| LF_3_30 | Mean | 164.430 | 17.128 |
|  | Median | 126.000 |  |
|  | Variance | 14960.930 |  |
|  | Std. Deviation | 122.315 |  |
|  | Minimum | 12.000 |  |
|  | Maximum | 515.000 |  |
|  | Range | 503.000 |  |
|  | Interquartile Range | 176.000 |  |
| HF_1_30 | Mean | 31.540 | 3.976 |
|  | Median | 26.500 |  |
|  | Variance | 758.934 |  |
|  | Std. Deviation | 27.549 |  |
|  | Minimum | 1.000 |  |
|  | Maximum | 134.000 |  |
|  | Range | 133.000 |  |
|  | Interquartile Range | 35.000 |  |
| HF_2_30 | Mean | 32.570 | 4.214 |
|  | Median | 27.000 |  |
|  | Variance | 834.554 |  |
|  | Std. Deviation | 28.889 |  |
|  | Minimum | 1.000 |  |
|  | Maximum | 120.000 |  |
|  | Range | 119.000 |  |
|  | Interquartile Range | 43.000 |  |
| HF_3_30 | Mean | 47.790 | 6.009 |
|  | Median | 40.000 |  |
|  | Variance | 1732.977 |  |
|  | Std. Deviation | 41.629 |  |
|  | Minimum | 1.000 |  |
|  | Maximum | 160.000 |  |
|  | Range | 159.000 |  |
|  | Interquartile Range | 44.000 |  |
| LF_1_60 | Mean | 224.940 | 21.418 |
|  | Median | 197.500 |  |
|  | Variance | 22936.139 |  |
|  | Std. Deviation | 151.447 |  |
|  | Minimum | 4.000 |  |
|  | Maximum | 705.000 |  |
|  | Range | 701.000 |  |
|  | Interquartile Range | 192.000 |  |
| LF_2_60 | Mean | 241.820 | 18.340 |
|  | Median | 225.000 |  |
|  | Variance | 16818.559 |  |
|  | Std. Deviation | 129.686 |  |
|  | Minimum | 12.000 |  |
|  | Maximum | 540.000 |  |
|  | Range | 528.000 |  |
|  | Interquartile Range | 157.000 |  |
| LF_3_60 | Mean | 255.500 | 20.020 |
|  | Median | 248.000 |  |
|  | Variance | 19238.638 |  |
|  | Std. Deviation | 138.703 |  |
|  | Minimum | 20.000 |  |
|  | Maximum | 700.000 |  |
|  | Range | 680.000 |  |
|  | Interquartile Range | 155.000 |  |
| HF_1_60 | Mean | 51.510 | 8.038 |
|  | Median | 37.000 |  |
|  | Variance | 3165.797 |  |
|  | Std. Deviation | 56.265 |  |
|  | Minimum | 1.000 |  |
|  | Maximum | 274.000 |  |
|  | Range | 273.000 |  |
|  | Interquartile Range | 60.000 |  |
| HF_2_60 | Mean | 51.710 | 7.249 |
|  | Median | 41.000 |  |
|  | Variance | 2574.583 |  |
|  | Std. Deviation | 50.740 |  |
|  | Minimum | 2.000 |  |
|  | Maximum | 235.000 |  |
|  | Range | 233.000 |  |
|  | Interquartile Range | 55.000 |  |
| HF_3_60 | Mean | 64.640 | 9.295 |
|  | Median | 54.000 |  |
|  | Variance | 4320.276 |  |
|  | Std. Deviation | 65.729 |  |
|  | Minimum | 1.000 |  |
|  | Maximum | 308.000 |  |
|  | Range | 307.000 |  |
|  | Interquartile Range | 80.000 |  |

| **Tests of Normality: Number of hits over 32dB** | | | |  |
| --- | --- | --- | --- | --- |
|  | Shapiro-Wilk | | | |
|  | Statistic | df | Sig. | |
| LF_1_30 | .931 | 51 | .005 | |
| LF_2_30 | .911 | 51 | <.001 | |
| LF_3_30 | .909 | 51 | <.001 | |
| HF_1_30 | .890 | 48 | <.001 | |
| HF_2_30 | .897 | 47 | <.001 | |
| HF_3_30 | .865 | 48 | <.001 | |
| LF_1_60 | .914 | 50 | .001 | |
| LF_2_60 | .936 | 50 | .010 | |
| LF_3_60 | .945 | 48 | .025 | |
| HF_1_60 | .784 | 49 | <.001 | |
| HF_2_60 | .810 | 49 | <.001 | |
| HF_3_60 | .826 | 50 | <.001 | |

| **Descriptive statistics: Number of hits over 36dB** | | | |
| --- | --- | --- | --- |
|  | | Statistic | Std. Error |
| LF_1_30 | Mean | 79.160 | 9.797 |
|  | Median | 56.000 |  |
|  | Variance | 4894.655 |  |
|  | Std. Deviation | 69.962 |  |
|  | Minimum | 2.000 |  |
|  | Maximum | 287.000 |  |
|  | Range | 285.000 |  |
|  | Interquartile Range | 108.000 |  |
| LF_2_30 | Mean | 95.290 | 11.722 |
|  | Median | 66.000 |  |
|  | Variance | 7007.532 |  |
|  | Std. Deviation | 83.711 |  |
|  | Minimum | 3.000 |  |
|  | Maximum | 354.000 |  |
|  | Range | 351.000 |  |
|  | Interquartile Range | 127.000 |  |
| LF_3_30 | Mean | 115.310 | 13.977 |
|  | Median | 90.000 |  |
|  | Variance | 9963.460 |  |
|  | Std. Deviation | 99.817 |  |
|  | Minimum | 6.000 |  |
|  | Maximum | 425.000 |  |
|  | Range | 419.000 |  |
|  | Interquartile Range | 151.000 |  |
| HF_1_30 | Mean | 19.960 | 3.150 |
|  | Median | 15.500 |  |
|  | Variance | 476.381 |  |
|  | Std. Deviation | 21.826 |  |
|  | Minimum | 1.000 |  |
|  | Maximum | 111.000 |  |
|  | Range | 110.000 |  |
|  | Interquartile Range | 20.000 |  |
|  | Skewness | 2.073 | 0.343 |
|  | Kurtosis | 5.550 | 0.674 |
| HF_2_30 | Mean | 19.450 | 3.036 |
|  | Median | 13.000 |  |
|  | Variance | 433.296 |  |
|  | Std. Deviation | 20.816 |  |
|  | Minimum | 1.000 |  |
|  | Maximum | 73.000 |  |
|  | Range | 72.000 |  |
|  | Interquartile Range | 24.000 |  |
| HF_3_30 | Mean | 29.690 | 4.402 |
|  | Median | 21.000 |  |
|  | Variance | 929.964 |  |
|  | Std. Deviation | 30.495 |  |
|  | Minimum | 1.000 |  |
|  | Maximum | 118.000 |  |
|  | Range | 117.000 |  |
|  | Interquartile Range | 33.000 |  |
| LF_1_60 | Mean | 136.060 | 16.332 |
|  | Median | 100.500 |  |
|  | Variance | 13337.078 |  |
|  | Std. Deviation | 115.486 |  |
|  | Minimum | 1.000 |  |
|  | Maximum | 594.000 |  |
|  | Range | 593.000 |  |
|  | Interquartile Range | 132.000 |  |
| LF_2_60 | Mean | 139.600 | 13.117 |
|  | Median | 119.500 |  |
|  | Variance | 8602.653 |  |
|  | Std. Deviation | 92.750 |  |
|  | Minimum | 7.000 |  |
|  | Maximum | 398.000 |  |
|  | Range | 391.000 |  |
|  | Interquartile Range | 104.000 |  |
| LF_3_60 | Mean | 151.810 | 14.146 |
|  | Median | 129.000 |  |
|  | Variance | 9604.794 |  |
|  | Std. Deviation | 98.004 |  |
|  | Minimum | 11.000 |  |
|  | Maximum | 438.000 |  |
|  | Range | 427.000 |  |
|  | Interquartile Range | 113.000 |  |
| HF_1_60 | Mean | 27.760 | 5.590 |
|  | Median | 15.000 |  |
|  | Variance | 1531.355 |  |
|  | Std. Deviation | 39.133 |  |
|  | Minimum | 1.000 |  |
|  | Maximum | 199.000 |  |
|  | Range | 198.000 |  |
|  | Interquartile Range | 30.000 |  |
| HF_2_60 | Mean | 28.510 | 5.155 |
|  | Median | 18.000 |  |
|  | Variance | 1302.047 |  |
|  | Std. Deviation | 36.084 |  |
|  | Minimum | 1.000 |  |
|  | Maximum | 151.000 |  |
|  | Range | 150.000 |  |
|  | Interquartile Range | 35.000 |  |
| HF_3_60 | Mean | 36.180 | 6.414 |
|  | Median | 21.000 |  |
|  | Variance | 2056.844 |  |
|  | Std. Deviation | 45.352 |  |
|  | Minimum | 1.000 |  |
|  | Maximum | 202.000 |  |
|  | Range | 201.000 |  |
|  | Interquartile Range | 47.000 |  |

| **Tests of Normality: Number of hits over 36dB** | | | |  |
| --- | --- | --- | --- | --- |
|  | Shapiro-Wilk | | | |
|  | Statistic | df | Sig. | |
| LF_1_30 | .888 | 51 | <.001 | |
| LF_2_30 | .880 | 51 | <.001 | |
| LF_3_30 | .871 | 51 | <.001 | |
| HF_1_30 | .784 | 48 | <.001 | |
| HF_2_30 | .817 | 47 | <.001 | |
| HF_3_30 | .815 | 48 | <.001 | |
| LF_1_60 | .845 | 50 | <.001 | |
| LF_2_60 | .906 | 50 | <.001 | |
| LF_3_60 | .893 | 48 | <.001 | |
| HF_1_60 | .685 | 49 | <.001 | |
| HF_2_60 | .737 | 49 | <.001 | |
| HF_3_60 | .763 | 50 | <.001 | |

| **Descriptive statistics: Mean number of hits per rotation** | | | |
| --- | --- | --- | --- |
|  | | Statistic | Std. Error |
| LF_1_30 | Mean | 12.056 | 0.960 |
|  | Median | 11.800 |  |
|  | Variance | 46.982 |  |
|  | Std. Deviation | 6.854 |  |
|  | Minimum | 0.950 |  |
|  | Maximum | 26.500 |  |
|  | Range | 25.550 |  |
|  | Interquartile Range | 10.250 |  |
| LF_2_30 | Mean | 13.041 | 1.072 |
|  | Median | 12.450 |  |
|  | Variance | 58.581 |  |
|  | Std. Deviation | 7.654 |  |
|  | Minimum | 0.900 |  |
|  | Maximum | 29.850 |  |
|  | Range | 28.950 |  |
|  | Interquartile Range | 11.300 |  |
| LF_3_30 | Mean | 14.802 | 1.246 |
|  | Median | 13.050 |  |
|  | Variance | 79.165 |  |
|  | Std. Deviation | 8.897 |  |
|  | Minimum | 1.300 |  |
|  | Maximum | 33.300 |  |
|  | Range | 32.000 |  |
|  | Interquartile Range | 15.900 |  |
| HF_1_30 | Mean | 18.575 | 1.672 |
|  | Median | 14.600 |  |
|  | Variance | 134.186 |  |
|  | Std. Deviation | 11.584 |  |
|  | Minimum | 4.600 |  |
|  | Maximum | 58.250 |  |
|  | Range | 53.650 |  |
|  | Interquartile Range | 10.910 |  |
| HF_2_30 | Mean | 21.633 | 2.122 |
|  | Median | 18.000 |  |
|  | Variance | 211.691 |  |
|  | Std. Deviation | 14.550 |  |
|  | Minimum | 4.600 |  |
|  | Maximum | 76.050 |  |
|  | Range | 71.450 |  |
|  | Interquartile Range | 13.150 |  |
| HF_3_30 | Mean | 24.576 | 2.221 |
|  | Median | 20.150 |  |
|  | Variance | 236.860 |  |
|  | Std. Deviation | 15.390 |  |
|  | Minimum | 6.950 |  |
|  | Maximum | 75.700 |  |
|  | Range | 68.750 |  |
|  | Interquartile Range | 14.960 |  |
| LF_1_60 | Mean | 13.381 | 0.980 |
|  | Median | 12.063 |  |
|  | Variance | 47.991 |  |
|  | Std. Deviation | 6.928 |  |
|  | Minimum | 0.500 |  |
|  | Maximum | 30.975 |  |
|  | Range | 30.475 |  |
|  | Interquartile Range | 11.788 |  |
| LF_2_60 | Mean | 14.107 | 0.912 |
|  | Median | 13.800 |  |
|  | Variance | 41.576 |  |
|  | Std. Deviation | 6.448 |  |
|  | Minimum | 0.700 |  |
|  | Maximum | 29.825 |  |
|  | Range | 29.125 |  |
|  | Interquartile Range | 8.881 |  |
| LF_3_60 | Mean | 14.568 | 1.071 |
|  | Median | 13.063 |  |
|  | Variance | 55.102 |  |
|  | Std. Deviation | 7.423 |  |
|  | Minimum | 1.275 |  |
|  | Maximum | 37.775 |  |
|  | Range | 36.500 |  |
|  | Interquartile Range | 7.625 |  |
| HF_1_60 | Mean | 15.121 | 1.139 |
|  | Median | 13.900 |  |
|  | Variance | 63.614 |  |
|  | Std. Deviation | 7.976 |  |
|  | Minimum | 3.525 |  |
|  | Maximum | 42.125 |  |
|  | Range | 38.600 |  |
|  | Interquartile Range | 10.200 |  |
| HF_2_60 | Mean | 17.458 | 1.399 |
|  | Median | 16.325 |  |
|  | Variance | 95.938 |  |
|  | Std. Deviation | 9.795 |  |
|  | Minimum | 4.025 |  |
|  | Maximum | 48.375 |  |
|  | Range | 44.350 |  |
|  | Interquartile Range | 13.213 |  |
| HF_3_60 | Mean | 21.141 | 1.667 |
|  | Median | 20.163 |  |
|  | Variance | 138.960 |  |
|  | Std. Deviation | 11.788 |  |
|  | Minimum | 5.400 |  |
|  | Maximum | 60.975 |  |
|  | Range | 55.575 |  |
|  | Interquartile Range | 15.769 |  |

| **Tests of Normality: Mean number of hits per rotation** | | | |  |
| --- | --- | --- | --- | --- |
|  | Shapiro-Wilk | | | |
|  | Statistic | df | Sig. | |
| LF_1_30 | .957 | 51 | .062 | |
| LF_2_30 | .951 | 51 | .034 | |
| LF_3_30 | .939 | 51 | .012 | |
| HF_1_30 | .859 | 48 | <.001 | |
| HF_2_30 | .837 | 47 | <.001 | |
| HF_3_30 | .833 | 48 | <.001 | |
| LF_1_60 | .962 | 50 | .105 | |
| LF_2_60 | .968 | 50 | .188 | |
| LF_3_60 | .949 | 48 | .037 | |
| HF_1_60 | .932 | 49 | .007 | |
| HF_2_60 | .907 | 49 | <.001 | |
| HF_3_60 | .924 | 50 | .003 | |
