# Supplementary material for: Correlation between proprioception, functionality, patient-reported knee condition and joint acoustic emissions
Source: PLoS One. 2024 Nov 6;19(11):e0310123. doi: 10.1371/journal.pone.0310123 (PMC11540232; doi:10.1371/journal.pone.0310123)
Supplement: S2 Appendix — (DOCX) [file pone.0310123.s002.docx]

| **Summary** | | | | | | |
| --- | --- | --- | --- | --- | --- | --- |
|  | Cases | | | | | |
|  | Valid | | Missing | | Total | |
|  | N | Percent | N | Percent | N | Percent |
| Age | 51 | 100.0% | 0 | 0.0% | 51 | 100.0% |
| Weight | 51 | 100.0% | 0 | 0.0% | 51 | 100.0% |
| Height | 51 | 100.0% | 0 | 0.0% | 51 | 100.0% |
| BMI | 51 | 100.0% | 0 | 0.0% | 51 | 100.0% |
| KOOS | 51 | 100.0% | 0 | 0.0% | 51 | 100.0% |
| KOOS_S | 51 | 100.0% | 0 | 0.0% | 51 | 100.0% |
| KOOS_P | 51 | 100.0% | 0 | 0.0% | 51 | 100.0% |
| KOOS_ADL | 51 | 100.0% | 0 | 0.0% | 51 | 100.0% |
| KOOS_SR | 51 | 100.0% | 0 | 0.0% | 51 | 100.0% |
| KOOS_QoL | 51 | 100.0% | 0 | 0.0% | 51 | 100.0% |
| 5STS | 48 | 94.1% | 3 | 5.9% | 51 | 100.0% |
| TTDPM | 48 | 94.1% | 3 | 5.9% | 51 | 100.0% |

| KOOS | Knee Injury and Osteoarthritis Outcome Score |
| --- | --- |
| KOOS_S | “Other Symptoms” KOOS subscale |
| KOOS_P | “Pain” KOOS subscale |
| KOOS_ADL | “Activities in Daily Living” KOOS subscale |
| KOOS_SR | “Function in Sport and Recreation” KOOS subscale |
| KOOS_QoL | “Knee-related Quality of Life” KOOS subscale |
| 5STS | 5 times sit-to-stand test |
| TTDPM | Threshold to detect passive motion |

# Abbreviations

| **Descriptive statistics** | | | |
| --- | --- | --- | --- |
|  | | Statistic | Std. Error |
| Age, years | Mean | 44.570 | 2.542 |
|  | Median | 50.000 |  |
|  | Variance | 329.530 |  |
|  | Std. Deviation | 18.153 |  |
|  | Minimum | 21.000 |  |
|  | Maximum | 75.000 |  |
|  | Range | 54.000 |  |
|  | Interquartile Range | 32.000 |  |
| Weight, kg | Mean | 75.887 | 2.112 |
|  | Median | 75.000 |  |
|  | Variance | 227.507 |  |
|  | Std. Deviation | 15.083 |  |
|  | Minimum | 48.000 |  |
|  | Maximum | 130.000 |  |
|  | Range | 82.000 |  |
|  | Interquartile Range | 18.000 |  |
| Height, cm | Mean | 172.381 | 1.262 |
|  | Median | 171.450 |  |
|  | Variance | 81.218 |  |
|  | Std. Deviation | 9.012 |  |
|  | Minimum | 155.000 |  |
|  | Maximum | 195.000 |  |
|  | Range | 40.000 |  |
|  | Interquartile Range | 15.000 |  |
| BMI, kg/m^2^ | Mean | 25.445 | 0.581 |
|  | Median | 25.030 |  |
|  | Variance | 17.212 |  |
|  | Std. Deviation | 4.149 |  |
|  | Minimum | 18.070 |  |
|  | Maximum | 37.980 |  |
|  | Range | 19.920 |  |
|  | Interquartile Range | 3.800 |  |
| KOOS | Mean | 83.370 | 2.075 |
|  | Median | 87.000 |  |
|  | Variance | 219.558 |  |
|  | Std. Deviation | 14.818 |  |
|  | Minimum | 47.000 |  |
|  | Maximum | 100.000 |  |
|  | Range | 53.000 |  |
|  | Interquartile Range | 22.000 |  |
| KOOS_S | Mean | 83.690 | 1.893 |
|  | Median | 89.000 |  |
|  | Variance | 182.740 |  |
|  | Std. Deviation | 13.518 |  |
|  | Minimum | 46.000 |  |
|  | Maximum | 100.000 |  |
|  | Range | 54.000 |  |
|  | Interquartile Range | 18.000 |  |
| KOOS_P | Mean | 88.160 | 1.887 |
|  | Median | 92.000 |  |
|  | Variance | 181.535 |  |
|  | Std. Deviation | 13.473 |  |
|  | Minimum | 42.000 |  |
|  | Maximum | 100.000 |  |
|  | Range | 58.000 |  |
|  | Interquartile Range | 19.000 |  |
| KOOS_ADL | Mean | 90.860 | 1.649 |
|  | Median | 96.000 |  |
|  | Variance | 138.641 |  |
|  | Std. Deviation | 11.775 |  |
|  | Minimum | 56.000 |  |
|  | Maximum | 100.000 |  |
|  | Range | 44.000 |  |
|  | Interquartile Range | 15.000 |  |
| KOOS_SR | Mean | 74.820 | 3.432 |
|  | Median | 85.000 |  |
|  | Variance | 600.788 |  |
|  | Std. Deviation | 24.511 |  |
|  | Minimum | 10.000 |  |
|  | Maximum | 100.000 |  |
|  | Range | 90.000 |  |
|  | Interquartile Range | 40.000 |  |
| KOOS_QoL | Mean | 79.550 | 2.923 |
|  | Median | 81.000 |  |
|  | Variance | 435.813 |  |
|  | Std. Deviation | 20.876 |  |
|  | Minimum | 19.000 |  |
|  | Maximum | 100.000 |  |
|  | Range | 81.000 |  |
|  | Interquartile Range | 31.000 |  |
| 5STS, s | Mean | 8.252 | 0.451 |
|  | Median | 7.905 |  |
|  | Variance | 9.765 |  |
|  | Std. Deviation | 3.125 |  |
|  | Minimum | 4.190 |  |
|  | Maximum | 19.830 |  |
|  | Range | 15.640 |  |
|  | Interquartile Range | 3.230 |  |
| TTDPM, deg | Mean | 1.241 | 0.195 |
|  | Median | 0.930 |  |
|  | Variance | 1.825 |  |
|  | Std. Deviation | 1.351 |  |
|  | Minimum | 0.250 |  |
|  | Maximum | 8.340 |  |
|  | Range | 8.090 |  |
|  | Interquartile Range | 0.700 |  |

| **Tests of Normality** | | | |
| --- | --- | --- | --- |
|  | Shapiro-Wilk | | |
|  | Statistic | df | Sig. |
| Age | .891 | 51 | <.001 |
| Weight | .957 | 51 | .064 |
| Height | .982 | 51 | .622 |
| BMI | .946 | 51 | .021 |
| KOOS | .899 | 51 | <.001 |
| KOOS_S | .879 | 51 | <.001 |
| KOOS_P | .816 | 51 | <.001 |
| KOOS_ADL | .779 | 51 | <.001 |
| KOOS_SR | .881 | 51 | <.001 |
| KOOS_QoL | .870 | 51 | <.001 |
| 5STS | .882 | 48 | <.001 |
| TTDPM | .566 | 48 | <.001 |
